# Lipoproteins in Gram-Positive Bacteria: Abundance, Function, Fitness

**DOI:** 10.3389/fmicb.2020.582582

**Published:** 2020-09-18

**Authors:** Minh-Thu Nguyen, Miki Matsuo, Silke Niemann, Mathias Herrmann, Friedrich Götz

**Affiliations:** ^1^Section of Medical and Geographical Infectiology, Institute of Medical Microbiology, University Hospital of Münster, Münster, Germany; ^2^Interfaculty Institute of Microbiology and Infection Medicine Tübingen (IMIT), Microbial Genetics, University of Tübingen, Tübingen, Germany

**Keywords:** lipoproteins, gram-positive bacteria, ion transport, host cell invasion, immune system

## Abstract

When one thinks of the Gram+ cell wall, the peptidoglycan (PG) scaffold in particular comes to mind. However, the cell wall also consists of many other components, for example those that are covalently linked to the PG: the wall teichoic acid and the cell wall proteins tethered by the sortase. In addition, there are completely different molecules that are anchored in the cytoplasmic membrane and span the cell wall. These are lipoteichoic acids and bacterial lipoproteins (Lpp). The latter are in the focus of this review. Lpp are present in almost all bacteria. They fulfill a wealth of different tasks. They represent the window to the outside world by recognizing nutrients and incorporating them into the bacterial cell via special transport systems. Furthermore, they perform very diverse and special tasks such as acting as chaperonin, as cyclomodulin, contributing to invasion of host cells or uptake of plasmids via conjugation. All these functions are taken over by the protein part. Nevertheless, the lipid part of the Lpp plays an as important role as the protein part. It is the released lipoproteins and derived lipopeptides that massively modulate our immune system and ultimately play an important role in immune tolerance or non-tolerance. All these varied activities of the Lpp are considered in this review article.

## Introduction

As far as we know today, bacterial lipoproteins (Lpp) are found in all bacteria. They are anchored in the membrane via their N-terminal lipid structure and are involved in a number of metabolic processes, some of which are vital. Using various computer-assisted algorithms, precursor Lpp can be identified by their signal peptide containing the characteristic “lipobox” (Babu et al., 2006). After cleavage of the signal peptide, Lpp are anchored into the bacterial membrane via a diacylglycerol moiety tethered via a thioether linkage to the N-terminal cysteine, and the free cysteine N-terminus is usually N-acylated (Buddelmeijer, 2015). The first Lpp to be elucidated was the Braun’s Lpp, a murein Lpp of *E. coli* which is anchored with its N-terminal lipid structure in the outer membrane of almost all Gram-negative bacteria (Hantke and Braun, 1973; Braun, 1975). As we now know, Lpp occur in all bacteria, and their structure, function and biosynthesis has been review recently (Sutcliffe and Russell, 1995; Braun and Hantke, 2019). In Gram+ bacteria, the Lpp’s lipid moieties are anchored in the outer layer of the cytoplasmic membrane and their protein portions span the cell wall. While the class of proteins covalently bound to the cell wall (peptidoglycan) via sortase consists mostly of adhesins such as immunoglobulin, fibronectin, or collagen binding proteins (Navarre and Schneewind, 1999), most Lpp are involved in nutrient uptake, possess enzymatic activities or fulfill very diverse and specific tasks. The biosynthetic steps from pre-Lpp to mature Lpp include two enzymatic reactions that appear to be highly conserved in all bacteria. These are diacylglyceryl transferase (Lgt) and the signal peptidase II (Lsp). A third modification, namely the acylation of the amino group of the N-terminal cysteine by the apolipoprotein N-acyltransferase (Lnt), occurs mainly in Gram-negative bacteria and the Gram+ actinobacteria. In firmicutes this third reaction is not compulsory and may be catalyzed by a different enzymatic reaction. The Lpp biosynthesis and the crucial role of Lpp maturation for virulence and TLR2 signaling have been recently described in detail (Buddelmeijer, 2015; Nguyen and Götz, 2016).

In this review, we analyze the total Lpp in 14 Gram+ bacteria species from different genera. In the first part, these Lpp are analyzed and classified by their function. In the second part, the diversity of lipid moiety structure in Gram+ Lpp and their impact on host immune modulation are discussed. The third part deals with the tightness of Lpp on the membrane and their release to the environment. Finally, we review the impact of Lpp as vaccine candidates.

## Function of Lpp in Gram+ Bacteria

Here we compared 14 bacterial species from 7 genera, i.e., *Staphylococcus*, *Bacillus*, *Listeria*, *Streptococcus*, *Enterococcus*, *Clostridium*, and *Mycobacterium* (Table 1). Most of the species play a role as pathogens. However, we have also included some non-pathogenic species from the *Bacillus* group in which some Lpp have been functionally well studied. The number of Lpp genes varies from species to species but accounts for 1–3% of all genes of a genome, representing considerable proportion of a bacterial genome.

**TABLE 1:** Number of Lpp of selected Gram-positive bacterial strains.

	Species	Strains	Org code	No of protein genes	No of Lpp	Description	References
Staphylococcus	*Staphylococcus aureus*	USA300	saa	2604	67	Round-shaped, non-spore forming, pathogenic bacterium. USA300 is a globally spread, highly virulent community MRSA strain.	Diep et al., 2006; Nimmo, 2012
Bacillus	*Bacillus subtilis*	subsp. subtilis 168	bsu	4174	63	Non-pathogenic, aerobic, endospore-forming, rod-shaped bacterium, commonly found in soil.	Kunst et al., 1997
	*Bacillus cereus*	ATCC 14579	bce	5231	96	Rod-shaped, anaerobic, spore forming bacterium. Foodborne pathogenic strain. Ubiquitous environmental distribution.	Ivanova et al., 2003
	*Bacillus licheniformis*	ATCC 14580	bli	4179	57	Rod-shaped, mesophilic, spore forming soil bacterium. Used in food industry.	Rey et al., 2004
	*Geobacillus kaustophilus*	HTA426	gka	3540	44	Rod-shaped, aerobic endospore forming bacterium. Isolated from the deep-sea sediment of the Mariana Trench.	Takami et al., 2004
	*Oceanobacillus iheyensis*	HTE831	oih	3496	99	Rod-shaped, aerobic endosporing forming bacterium. An alkaliphilic and extremely halotolerant Bacillus-related species isolated from a deep-sea sediment collected at a depth of 1050 m on the Iheya Ridge, Japan.	Takami et al., 2002
Listeria	*Listeria monocytogenes*	EGD-e	lmo	2867	53	Foodborne pathogen, non-spore forming bacteria. The serotype 1/2a strain tends to be more sporadic and not associated with epidemics.	Glaser et al., 2001; Donaldson et al., 2009
Streptococcus	*Streptococcus pneumoniae*	TIGR4	spn	2125	27	Pathogenic, non-spore forming, facultative anaerobic and round-shape bacterium. Major cause of bacterial pneumonia and other invasive infection.	Tettelin et al., 2001
	*Streptococcus pyogenes*	M1 GAS	spy	1693	26	Pathogenic, aerotolerant, non-spore forming, and round shape bacterium, frequently causing skin and soft tissue infections.	Ferretti et al., 2001
	*Streptococcus agalactiae*	NEM361	san	2094	27	Pathogenic, facultative anaerobic and round-shape bacterium. Group B streptococcus (GBS), frequent colonizer of the urogenital tract and major cause of neonatal sepsis.	Glaser et al., 2002
	*Streptococcus mutans*	UA159	smu	1960	20	Pathogenic, anaerobic and round shape bacteria. Part of the oropharyngeal flora, major cause for dental caries. Potential for invasive disease (endocarditis).	Ajdic et al., 2002
Enterococcus	*Enterococcus faecalis*	V583	efa	3264	59	Opportunistic pathogenic, anaerobic, non-motile, round-shape bacterium. Vancomycin-resistant clinical isolate from blood cultures from an indexed patient in the United States in 1987.	Paulsen et al., 2003
Clostridium	*Clostridium perfringens*	13	cpe	2723	36	Rod-shaped, anaerobic, spore-forming pathogenic bacterium. Cause of gas gangrene.	Shimizu et al., 2002
Mycobacterium	*Mycobacterium tuberculosis*	CDC1551	mtc	4189	30	Obligate pathogenic, aerobic bacterium, acid-fast. Causative agent of tuberculosis in humans and animals.	Fleischmann et al., 2002

**FIGURE 1:**
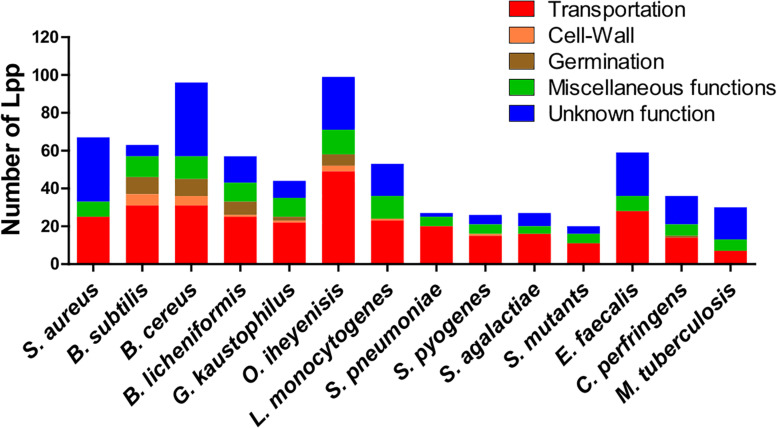
Lipoproteins content in 14 Gram+ bacterial species. Lpp are categorized by their function such as transportation, cell-wall turnover, germination, miscellaneous functions and unknown function. A more detailed information of Lpp in each group are presented in Supplementary Table S1–S13 for 13 species (*Bacillus subtilis*, *Bacillus cereus*, *Bacillus licheniformis*, *Geobacillus kaustophilus*, *Oceanobacillus iheyenisis*, *Listeria monocytogenes*, *Streptococcus pneumoniae*, *Streptococcus pyogenes*, *Streptococcus agalactiae*, *Streptococcus mutants*, *Enterococcus faecalis*, *Clostridium perfringens*, and *Mycobacterium tuberculosis*). Data for *Staphylococcus aureus* Lpp are taken from Shahmirzadi et al. (2016).

Some genera contain a relatively high, others a comparatively low number of Lpp (20 to 35), such as streptococci, *C. perfringens* and *M. tuberculosis* (Figure 1). The high number of different Lpp and also the high expression of certain Lpp such as MntC (SitC)-binding protein of a manganese ABC transporter (Stoll et al., 2005) show that this class of proteins can make up a major part of the cell wall associated proteins. However, their impact on peptidoglycan biosynthesis and structure are barely investigated.

### Substrate Binding in ABC Transport System

In Table 2 the diverse Lpp are summarized and grouped according to their function and occurrence in the selected bacterial species. The full list of Lpp genes in the various Gram+ bacteria with the annotated function is described in Supplementary Tables S1–S13. A large proportion of Gram+ Lpp are substrate-binding proteins (SBPs) of ABC transporter systems responsible for the acquisition of multiple nutrients including amino acids and short peptides, sugars, polyamines, and many metal ions. Some of the transporter activities are described below in more detail.

**TABLE 2:** Number of lipoprotein genes categorized as function.

Function	*S. a*	*B. s*	*B. c*	*B. l*	*G. k*	*O. i*	*L. m*	*S. pneu*	*S. pyro*	*S. a*	*S. m*	*E. f*	*C. p*	*M. t*
**Transportation**	25	31	31	25	22	49	23	20	15	16	11	28	14	7
Fe	8	5	8	5	3	4	4		1			3	1	
Zn, Mo, Mn….	6	5				3	3	2	2	1		3		1
PO4, NO3….	3	2		2	2	3		1	1	2	1	2	1	1
AA and oligopeptide	7	10	20	6	8	23	8	8	3	4	5	13	4	2
Sugar	1	6	2	7	2	6	5	2	3		2	2	3	1
Lipid														2
Unknown transp.		3	1	5	7	10	3	7	5	9	3	5	5	
**Cell-Wall**		6	5	1	1	3	1		1					
Penicillin-binding protein, transpeptidase		3	2			1								
Carboxypeptidase					1		1							
Polysaccharide deacetylase		1	1											
YqiH		1		1										
LytA		1												
Succinoglycan biosynthesis			1											
Amidase						1								
Ppiase B						1								
Peptidoglycan hydrolase									1					
Capsule biosynthesis			1											
**Germination**		9	9	7	2	6							1	
**Enzymes and foldases**	8	11	12	10	10	13	12	5	6	4	5	8	6	6
Membrane insertase YidC	1	1	2	1	2	1	2	1	1	2	2	1		
Peptidylprolyl isomerase PrsA	1		4	1	1	1	2	1	1	2	1	1		
Peptidylproly isomerase B								1	1		1	1	1	
Superoxide dismutase (SOD)		1			1	1								1
SCO family protein			1		1	1								
Thioredoxin family	1							1					1	1
Respiratory chain	1	2		2	1									
Beta lactamase	1													
Proteinase/Peptidase						1		1	1		1	1		1
Hydrolase/Esterase/Lipase		2	2			2	3					1		
Phosphatase			1						1			1	1	1
Nisin, subtilin immunity (LanI)		1												
CamS family sex pheromone	1	1	1			1	1							
Other enzymes	2	3	1	6	4	5	4		1			2	3	2

#### Fe-Acquisition

A relatively high proportion of Lpp is involved in ion transport. Bacterial pathogens in particular employ numerous strategies to acquire the essential nutrient iron since the amount of free iron in the host is extremely low. Several of the Fe-acquisition system involves Lpp. For instance, in *S. aureus* USA300 8 Lpp are associated with iron acquisition or utilization of host-derived heme iron (isd operon) as an iron source (Shahmirzadi et al., 2016). One of the more intensively studied iron transporter systems in *S. aureus* uses the two Lpp (FhuD1 and FhuD2) which are part of the FhuCBG system (Sebulsky et al., 2000). FhuD1 and FhuD2 bind iron(III)-hydroxamate siderophores and present it to the ABC transporter FhuCBG. Another well studied iron transporter system is SirABC which is a more promiscuous importer transporting ferric hydroxamates, ferric enterobactin or ferric citrate and staphylobactin (Dale et al., 2004). Other transport systems initially stirring great interest among bacterial physiology researcher are the twin-arginine translocation (TAT) systems in pathogenic staphylococci. But the reality did not meet fully expectations. In fact, only the existence of one TAT system, namely FepABC could be confirmed comprising of the Lpp FepA domain representing the Fe-binding protein, FepB with the TAT signal peptide functioning as an Fe-dependent peroxidase, and FepC moiety presumably acting as the Fe transporter through the membrane (Biswas et al., 2009).

In staphylococci there is a correlation between the amount of Lpp-dependent Fe-uptake systems and pathogenicity; increasing pathogenic potential appears to be paralleled by an enhanced number of Lpp-dependent Fe-uptake systems (Shahmirzadi et al., 2016). There are only three bacterial groups where Lpp-mediated Fe-transporters are either absent or not yet identified: streptococci and *M. tuberculosis* (Table 2). This is astonishing, as iron is an essential nutrient for the growth of most bacteria. The exceptions are certain lactic acid bacteria that do not need iron since they use manganese instead (Götz et al., 1980; Archibald, 1986). But most other bacteria have developed specific iron-transport systems located on the membrane surface to take up iron and iron complexes such as heme or ferrichrome. *S. pneumoniae* uses ferrichrome as an iron source (Yang et al., 2014). To reveal the reason of lacking Lpp-dependent Fe transport system, we blasted the Fe-binding *lpp* genes in streptococci, and found the signal peptide of *lpp* genes modified into transmembrane signal peptide. *M. tuberculosis* uses the ESX-3 secretion system (Type VII secretion systems, T7SS) which is essential for siderophore-mediated iron uptake and for heme utilization (Zhang et al., 2020); this system does not involve Lpp. This might explain why *M.t.* does not involve an Lpp in iron acquisition.

**FIGURE 2:**
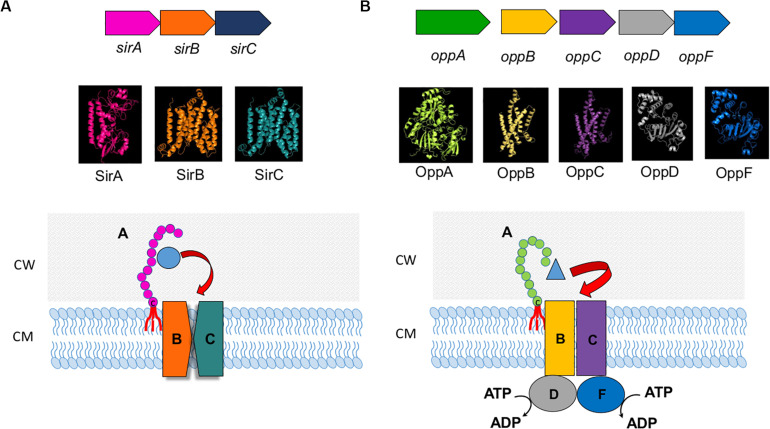
ABC transport system. **(A)** sirABC ion transport system. Upper panel illustrates the operon in *Staphylococcus aureus* (Dale et al., 2004). Middle panel presents 3D models of SirA, SirB, and SirC. 3D crystal model of SirA was downloaded from protein data bank (PDB ID: 3MWF) and visualized in PyMoL software (DeLano, 2009). Phyre2 (Kelley et al., 2015) was used to predict the 3D configuration of SirB and SirC. The Phyre2 predition showed 8 transmembrane motifs in both SirB and SirC ranging from 14 to 324 amino acids in SirB and 16 to 326 amino acids in SirC. There were 2 re-entrant helices which connect the 5th to 6th and 6th to 7th transmembrane motifs in both SirB and SirC. Superimposition of SirB to SirC in PyMoL showed high similarity in the 3D structure of SirB and SirC. The lower panel is the illustration of the SirABC transport system for Fe-staphylobactin (green circle). **(B)** OppABCDF oligopeptide transport system. The upper panel illustrates the operon in *Bacillus subtilis* (Perego et al., 1991). Middle panel presents 3D models of OppA, B, C, D and F. Phyre2 (Kelley et al., 2015) was used to predict the 3D configuration of OppA, OppB, OppC, and OppD. 3D crystal model of OppF was downloaded from protein data bank (SMR ID: P24137) and visualized in PyMoL software (DeLano, 2009). The Phyre2 predition showed 6 transmembrane motifs in both OppB and OppC ranging from 6 to 297 amino acids in OppB and 43 to 291 amino acids in OppC. Superimposition of OppB and OppD to OppC and OppF in PyMoL showed high similarity in the 3D structure of OppB and OppC, and OppD and OppF, respectively. The lower panel is the illustration of the OppABCDF transport system for oligopeptide (blue triangle).

In *S. pyogenes* the Lpp MtsA as part of the MtsABC iron transporter system binds primarily Fe(2+) > Fe(3+) > Cu(2+) > Mn(2+) > Zn(2+) (Sun et al., 2008, 2009). Interestingly, MtsA requires bicarbonate as a synergistic anion for stable ferrous binding which is similar to the iron binding in human transferrin. Another Fe-containing Lpp in *S. pyogenes* is SiaA, part of the SiaABC heme transporter (Sun et al., 2010). A typical ABC transport system with Lpp as a substrate binding protein being part of it is shown in Figure 2A for the sirABC system in *S. aureus* (Dale et al., 2004).

#### Other Cation Transporters

Other cation transporters mediating the transport of Co, Cu, Mn, Mo, Ni, Zn ions and often involving Lpp have also been studied. Similar to Fe, many of these ions are limited in the infection environment. Therefore, these transporters are very important for survival in an infection, hence also for the pathogenicity of the respective bacteria. Especially Mn plays an important role besides Fe. It is therefore not surprising that one of the most frequently represented Lpp in *S. aureus* is the Mn-binding protein (MntC) (Stoll et al., 2005). It was formerly called SitC because it was thought to be a Fe binding protein, but MntABC turned out to be a Mn transport system (Horsburgh et al., 2002; Diep et al., 2014).

Such manganese transporters with MntA homologs are also prevalent in other bacteria such as bacilli (MntA) or streptococci (PsaA) (Bray et al., 2009; Li et al., 2014). In *S. aureus* three Lpp were annotated as Ni transporters. The *S*. *aureus* Cnt (Opp1) ABC transporter imports nickel and cobalt in zinc-depleted conditions and contributes to virulence (Remy et al., 2013). The Lpp ModA is part of the molybdate transporter complex (ModABC) (Neubauer et al., 1999). In Group A *Streptococcus* (GAS) the two surface Lpp, AdcA and AdcAII, are involved in zinc uptake and zinc homeostasis with AdcA acting as a zinc-specific importer (Tedde et al., 2016). Deletion of the zinc transporter Lpp AdcAII caused hyperencapsulation of *S. pneumoniae* associated with distinct alleles of the type I restriction-modification system (Durmort et al., 2020).

#### Anion Transporters

Lipoproteins provide functions as various anion transporters for phosphate, phosphonate or nitrate. In bacilli Lpp are involved in the osmo-regulated ABC transport system OpuA (Kempf et al., 1997). This is a high-affinity uptake system for the osmoprotectant glycine betaine which consists of ATPase (OpuAA), an integral membrane protein (OpuAB), and the Lpp (OpuAC).

#### Amino Acid and Peptide Transporters

Amino acid and peptide transporters involving Lpp are even more abundant than Fe-transporters. One of the first described oligopeptide permease involving an Lpp is the OppABCD(F) system (Figure 2B), in which the OppA represents the Lpp. OppB and OppC are integral membrane proteins, and OppD and OppF are ATP binding proteins that couple ATP hydrolysis to the transport process (Perego et al., 1991). OppA variants are involved in surfactin regulation in *B. subtilis* (Wang et al., 2019). In *Bacillus* there are more such oligopeptide transporters as for example the AppDFABC with AppA corresponding to binding oligopeptide (Koide and Hoch, 1994). In *Staphylococcus aureus* 7 Lpp were identified being involved in amino acid and oligopeptide transport. One of the most important systems seems to be Opp3 (Hiron et al., 2007) in relevance followed by GmpC which is part of an ABC transporter and binds glycyl-methionine (Williams et al., 2004). Methionine is one of the least abundant amino acids in physiological fluids (4 μg ml^–1^) and many bacteria such as streptococci possess an ABC transporter (MetQNP) for methionine uptake. MetQ represents the Lpp that binds methionine (Basavanna et al., 2013); this transporter is crucial for growth and virulence.

#### Sugar Transporters

It has been known that sugars are transported by the phosphotransferase system (PTS). Only few studies relating sugar ABC transporters are available. However, screening the Lpp database we found a number of Lpp genes are responsible for sugar binding in ABC transport system (Table 2). It has been shown that the maltodextrin uptake is related to ABC transporter during host colonization by *E. faecalis* (Sauvageot et al., 2017).

#### Lipid Transporter

Lipid transporters that involve Lpp are rare in bacteria with the exception of mycobacteria. *M. tuberculosis* imports cholesterol across its atypical diderm cell envelope. Four closely related ATP-binding cassette (ABC) transporter-like complexes called Mce are responsible for the import (Wilburn et al., 2018). Three of the Mce proteins represent Lpp (Fenn et al., 2019). Mce proteins are involved in modulating host cell signaling and allow the growth with cholesterol as sole C-source.

#### Cell Wall

Little is known about Lpp involved in cell wall biosynthesis or degradation. In *Bacillus* spp., *Lactobacillus* spp. and *Staphylococcus* spp. an Lpp named New Lipoprotein C/Protein of 60-kDa (NlpC/P60) has been identified. This is a papain-like cysteine peptidase involved in the catalysis of the N-acetylmuramate-L-alanine or D-γ-glutamyl-meso-diaminopimelate linkages (Anantharaman and Aravind, 2003; Vermassen et al., 2019). In *Bacillus* group, Lpp has been annotated as putative polysaccharide deacetylase, cell elongation specific DD-transpeptidase, and penicillin-binding proteins, respectively (Supplementary Tables S1–S13). The gene cluster YqiHIK, with YqiH as an Lpp is involved in hydrolytic activity of the peptidoglycan saccule. Even though YqiHIK is not critical for reestablishing the rod-shaped morphology, the deletion of this operon impaired growth in a defined minimal medium (Fischer and Bremer, 2012). Moreover in *Bacillus* spp. the operon lytRABC with lytA as Lpp regulates the gene of N-acetylmuramoyl-L-alanine amidase (Lazarevic et al., 1992).

#### Spore Germination

In group of rod shape bacteria producing endospores, Lpp genes are also involved in the spore germination process for example GerA/D in *Bacillus subtilis* or GerS in *Clostridium difficile* (Pelczar and Setlow, 2008; Li et al., 2014; Diaz et al., 2018). In fact, a *Bacillus anthracis lgt* mutant lacking the lipid moiety germinated inefficiently; in line, the spore displayed attenuated virulence in the mouse infection model (Okugawa et al., 2012).

### Enzymes and Foldases

#### YidC

YidC is probably the most important and also essential Lpp in bacteria. It has been first functionally analyzed in *E. coli* as a membrane insertase for Sec-independent proteins (Samuelson et al., 2000; Serek et al., 2004). The *E. coli* YidC is a 61 kD protein of the inner (cytoplasmic) membrane and is composed of 5–6 transmembrane (TM) helices that contact hydrophobic segments of the substrate proteins. Since YidC also cooperates with the SecYEG translocon it is widely involved in the assembly of many different membrane proteins including proteins that obtain complex membrane topologies (Petriman et al., 2018). YidC homologs are not only present and essential in bacteria but homologs are also found in mitochondria (Oxa1) and thylakoids (Alb3) a fact pointing toward a common evolutionary origin, and also demonstrating the general importance of this cellular process (Dalbey et al., 2014). It is therefore not surprising that YidC homologs are present in all bacteria.

#### Peptidylprolyl Isomerase PrsA

PrsA homologs are also very abundant in bacteria. In most bacteria it is an essential membrane-bound Lpp that is involved in protein secretion (Kontinen and Sarvas, 1993). Prolyl *cis*/*trans* isomerase (PPIases) PrsA accelerates the folding of proteins containing *cis*-prolines thus abrogating the rate-limiting steps in the folding of proteins containing *cis*-prolines (Gothel and Marahiel, 1999). Numerous reports have demonstrated that *prsA* mutants are affected in protein secretion, and also in virulence in diverse Gram+ pathogens (Vitikainen et al., 2004; Heikkinen et al., 2009; Guo et al., 2013; Chen et al., 2015; Jousselin et al., 2015; Liu et al., 2019).

#### Respiratory Chain

In *Bacillus* spp. at least one Lpp, i.e., the SCO (Synthesis of Cytochrome c Oxidase) plays a role in the respiratory chain. SCO homologs are found in many bacteria. They have a high affinity for Cu^2+^ and are required for the proper synthesis of cytochrome c oxidase (Xu et al., 2015). Moreover, in *S. aureus* QoxA is an Lpp. QoxA is part of the terminal cytochrome aa3 quinol oxidase encoded by *qoxABCD* (Götz and Mayer, 2013; Hammer et al., 2013). In *S. pneumonia* two surface-exposed thioredoxin-family lipoproteins, Etrx1 and Etrx2, are crucial for resistance to H_2_O_2_ oxidative stress (Saleh et al., 2013).

#### Enzymes

A considerable number of Lpp have been shown to exert enzymatic activity. Many Gram+ pathogens contain two classes of Lpp molecules conferring ß-lactamase activities, a secreted and a membrane bound Lpp. Such Lpp-penicillinases are described in *B. licheniformis*, *B. cereus*, and *S. aureus* (Nielsen and Lampen, 1982a,b). The *S. aureus* Lpp-penicillinase was one of the first Lpp described in a Gram+ bacterium. Other Lpp enzymes have peptidase, protease, lipase, esterase or phosphatase activities. For example, the LppC of *Streptococcus equisimilis* functions as an acid phosphatase (Malke, 1998). In various mycobacteria three Lpp with phosphatase activity (PhoA, SapM) were described (Wolschendorf et al., 2007). Phosphorus is indispensable for the biosynthesis of nucleic acids and phospholipids and for the energy supply of any cell.

#### Lantibiotic Immunity Lpp (LanI)

Lantibiotics are a subgroup of bacteriocins from Gram+ bacteria consisting of polycyclic peptides containing modified amino acids. The biosynthetic gene cluster of Pep5, Epicidin 280, nisin and subtilin contain an immunity gene that encodes a small Lpp collectively named LanI (Hoffmann et al., 2004; Geiger et al., 2019). The activity of LanI molecules on lantibiotics is highly specific. For example, NisI protects the bacterial cell against nisin, but not against structurally very similar lantibiotics from other species such as subtilin from *B. subtilis*. Structural analysis reveals that the LanI’s C-terminal domain binds nisin, thereby preventing nisin from reaching its target molecules (Hacker et al., 2015; Jeong and Ha, 2018).

#### Sex Pheromones

*Enterococcus faecalis*, *S. aureus*, *S*. *epidermidis*, *B*. *subtilis*, and *L*. *monocytogenes* produce sex pheromones which are hydrophobic heptameric or octameric peptides derived from the C-terminal Lpp signal sequences (Flannagan and Clewell, 2002; Chandler and Dunny, 2004). These sex pheromones act as signals that facilitate the conjugative transfer of a specific category of plasmids referred to as pheromone-responsive plasmids (Wirth, 1994; Dunny and Leonard, 1997). Eep, a zinc-dependent membrane metalloprotease, has been shown to be involved in processing the Lpp precursor, leading to production of the active pheromone in most but not all pheromone systems (Chandler and Dunny, 2008).

### Lpl Are a Special Class of Lpp Involved in Invasion

In *S. aureus* a special class of Lpp has been described that contributes to host cell invasion (Nguyen et al., 2015). These Lpp are called “lipoprotein-like” lipoproteins (Lpl). The corresponding genes are organized in tandem on a pathogenicity island. It was shown that Lpl induce invasion by host cells, the invasion process being mediated by the protein portion and not by the lipid moiety of Lpl (Nguyen et al., 2015). The increased invasion in murine skin and an increased bacterial burden in a murine kidney abscess model suggest that the lpl gene cluster serves as an important virulence factor. The protein part of Lpls’ exerts a number of activities on host cells. One activity is that they delay G2/M phase transition in HeLa cells thus acting as cyclomodulins (Nguyen et al., 2016). Unlike the other staphylococcal cyclomodulins, Lpl1 (the model Lpl) shows no cytotoxicity even at high concentrations. A high conservation among Lpl proteins might be suggestive for common function. These observations may stir a speculation that a link exists between the exposure of Lpls on the bacterial surface and the delay in eukaryotic G2/M phase transition delay ultimately influencing host cell invasion processes.

In fact, the underlying mechanism of Lpl-triggered host cell invasion has been recently elucidated (Tribelli et al., 2019). The Lpl1 protein part, without the lipid moiety, binds directly to the isoforms of the human heat shock protein Hsp90-alpha and Hsp90-beta (Tribelli et al., 2019). Although the Hsp90-beta is constitutively expressed, the Hsp90-alpha isoform is heat-inducible and appears to play a major role in Lpl1 interaction. Lpl1-Hsp90 interaction induces F-actin formation, thus triggering an endocytosis-like internalization. The Lpl induced internalization represents a new host cell invasion principle (Tribelli et al., 2019). Pre-incubation of HaCaT cells at 39°C increased both the Hsp90-alpha expression and *S. aureus* invasion. It may be assumed that evolutionary adaption of *S. aureus* to the host has provided for protective mechanism of the microorganism against elevated temperatures in the host, i.e., fever. Above observation, namely the Lpl1/Hsp90-alpha mediated F-actin formation, now allows for the exciting hypothesis that enhanced bacterial invasion might provide a cellular niche for the microorganisms thus protection from host defense. Interestingly, Lpl proteins are mainly found in *S. aureus* and not in other staphylococcal species or other genera (Shahmirzadi et al., 2016).

Taken together, some Lpp are much conserved and present in almost all bacterial species. However, each species, and even each strain, contains its own specific Lpp, depending on its genetic make-up and habitat. The size of Lpp varies from <20 kDa up to >60 kDa which correlates with their function (Table 3). Some Lpp with many membrane spanning domains and some external and cytoplasmic loops like YidC are mainly localized in the membrane, while most others penetrate and span the cell wall and are exposed to the surface of the bacteria. Our knowledge about the function of Lpp in bacteria is incomplete and still about 30% of the Lpp have unknown functions, and to present it is unclear whether some of them are at all essential.

**TABLE 3:** The correlation between the function and the size of Lpp.

Catalog Lpp by size	Function	Proteins
**Large** (more than 40 kDa)	**Transportation**	Peptide/oligopeptide transp. (≥50 kDa)
		Sugar transp. (40–50 kDa)
	**Cell-wall**	penicillin-binding protein
	**Miscellaneous**	Phosphatase (40 kDa), Copper resistant protein A (50 kDa),
**Medium** (20–30 kDa)	**Transportation**	Fe, other cation, anion and amino acid transp. (25–35 kDa)
	**Germination**	Spore germination protein
	**Cell wall**	Enzymes
	**Miscellaneous**	YidC, chaperon, foldase, peptidylprolyl isomerase, oxidase, hydrolase, nuclease, regulator of comK, CamS family sex pheromone protein
**Small** (<20 kDa)	**Cell-wall**	YqiH, LytA,
	**Miscellaneous**	Thioredoxin family protein, inhibitor, SCO family, cytochrome, superoxide dismutase

## Lpp Lipid Structure and Immunity Activity

Unlike other proteins, Lpp contains an unusual ether linked S-glyceryl-cysteine residue modified with three fatty acids (N-acyl-S-diacylglyceryl cysteine) at its N-terminus. This unique N-terminal lipid structure anchors Lpp in the bacterial membrane.

**FIGURE 3:**
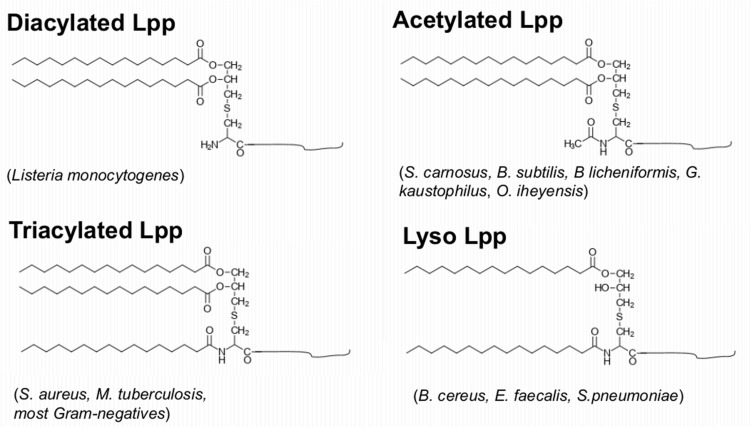
Variation of the Lpp’s lipid structures in Gram+ bacteria. Diacylated Lpp contain a modified S-diacyl-glyceryl cysteine which is found for example in *Listeria monocytogenes*; triacylated Lpp contain a modified N-acyl-S-diacyl-glyceryl cysteine which were found in *S. aureus*, *M. tuberculosis*, and most Gram-negatives; N-acetylated Lpp contain N-acetyl-S-diacyl-glyceryl cysteine which were found in *S. carnosus*, *B. subtilis*, *B. licheniformis*, *G. kaustophilus*, *O. iheyensis*; Lyso Lpp contain a modified N-acyl-S-monoacyl cysteine which were found in *B. cereus*, *E. faecalis*, or *S. pneumoniae*.

In Gram+ bacteria Lpp and/or lipopeptides are the main TLR2 agonists and they play a similar role as the lipopolysaccharides (LPS) in Gram-negative bacteria. In our previous review we raised the impact of Lpp in bacterial virulence and their function as TLR2 ligand (Nguyen and Götz, 2016). Here we concentrate on how Gram+ bacteria alter their lipid structures to modulate the host immune system. Gram-negative bacteria contain tri-acylated Lpp, however, the structure of the lipid moiety of Gram+ Lpp may vary from species to species (Figure 3).

### N-Acetylation and Lyso-Form in Firmicutes

The enzymes involved in modification and processing of the Lpp in *E. coli* are well studied (Buddelmeijer, 2015). The third and last modification step is the N-acylation of diacylglyceride-cysteine by apolipoprotein N-acyltransferase (Lnt), resulting in mature N-acyl-S-diacylglyceryl-cysteine linked proteins (Buddelmeijer, 2015). While Lgt and Lsp are conserved in all bacterial species, Lnt has only been identified in proteobacteria and actinomycetes. Therefore, it has been assumed that in low GC Gram+ bacteria (e.g., firmicutes) Lpp are only diacylated (Stoll et al., 2005). However, with the development of GC-MS technology (Kurokawa et al., 2012) showed that Lpp from Gram+ bacteria may have different lipid structures. Diacylated Lpp with two O-acylated long-chain fatty acids are described in *L. monocytogenes* (Kurokawa et al., 2012). Triacylated Lpp with the N-terminal cysteine being N-acylated with a long chain fatty acid is described in *S. aureus*, *M. tuberculosis* and in most Gram-negative bacteria (Kurokawa et al., 2009; Tschumi et al., 2009; Buddelmeijer, 2015). An N-acetylated lipid structure was found in *S. carnosus*, *B. subtilis*, *B licheniformis*, *G. kaustophilus*, and *O. iheyensis* (Kurokawa et al., 2012; Kumari et al., 2017). Finally, Lyso-Lpp structures that lack the O-acyl group in A-2 position have been found in *B. cereus*, *E. faecalis* and *S. pneumoniae* (Kurokawa et al., 2012; Armbruster and Meredith, 2017).

These observations raise the important question how firmicutes which do not process a Lnt homolog carry out N-acylation of their Lpp. Recently, it was found that in *S. aureus* two genes/enzymes are involved in N-acylation of Lpp (Gardiner et al., 2020). They are referred to as N-acylation transferase system (Lns). LnsA is an NlpC/P60 superfamily enzyme while LnsB has remote homology to the CAAX protease and to the bacteriocin-processing enzyme (CPBP) family. With either both LnsA and LnsB being necessary, or one enzyme alone being sufficient for N-acylation in *S. aureus*, they convert the Lpp chemotype from the diacylated to the tri-acylated form when heterologously expressed in *L. monocytogenes*.

**FIGURE 4:**
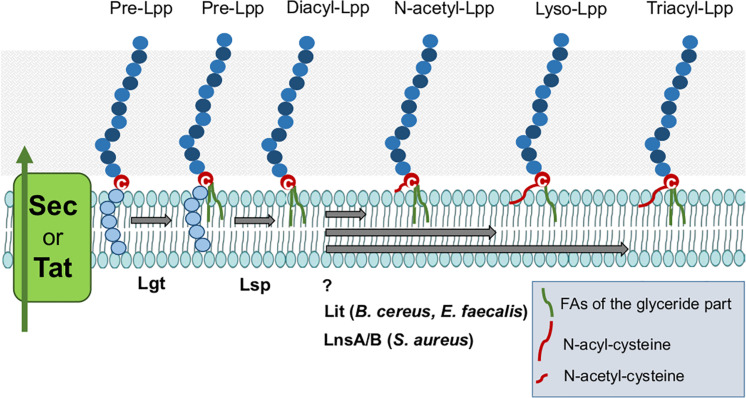
Lipoproteins biosynthesis modification in Firmicutes. After Sec/Tat dependent export, precursor Lpp are located on the cytoplasmic membrane where they can be modified by processing enzymes to yield the mature Lpp. The first enzyme Lgt transfers the diacylglyceryl group from phosphatidylglycerol to the sulfhydryl group of the invariant cysteine residue in the lipobox. The second enzyme Lsp recognizes the diacylglyceryl modified lipo signal peptide and cleaves between the amino acid at position –1 and the lipid-modified cysteine residue at +1, obtaining the diacyl-Lpp. N-acetyl-Lpp are modified by unknown enzyme. Lyso-Lpp are modified by Lit enzyme. And Tri-acyl (N-acyl)-Lpp are processed by LnsAB in *S. aureus*.

As shown in Figure 3 *E. faecalis* and *B. cereus* produce Lyso-Lpp. The Lyso-Lpp is created by a new enzyme, the Lpp intramolecular transacylase (Lit) (Armbruster and Meredith, 2017). The discovery of lipid modifying enzymes in Lpp of Gram+ bacteria is an important step forward in analyzing the consequences of the various lipid structures for the immune modulation. The biosynthesis modification of Lpp in firmicutes is illustrated in Figure 4.

### Gram+ Bacteria Differ in the Lipid Structure and Immune Modulation

In recent years it has been shown that the lipid structure exerts a tremendous influence on the immune response both of the innate and the adaptive system. A number of studies have been conducted with various staphylococcal species, the majority being skin residents and commensals, while others, like *S. aureus* may cause infections and abscesses. In a study on cutaneous innate immune sensing diacylated and triacylated lipopeptides were compared (Skabytska et al., 2014). In a mouse model diacylated Lpp was shown to induce a much higher inflammatory response than triacylated Lpp. On the other hand through the massive production of interleukin-6 (IL-6), diacylated Lpp induced the activation Gr1(+)CD11b(+) myeloid-derived suppressor cells (MDSCs) which are recruited to the skin. The recruited MDSCs suppress T cell-mediated recall responses such as dermatitis thus dampening the overshooting immune reaction. Therefore, certain Lpp on the skin may counteract inflammation by suppressing immune responses via activation of MDSCs (Skabytska et al., 2014). This study is one of the first reports showing that the different lipid structures of Lpp induce either high or low immune stimulation.

In fact, this insight has recently showed when comparing the immune response of the commensal *S. aureus* and *S. epidermidis* with that of the non-commensal *S. carnosus* species (Nguyen et al., 2017). Structural analysis of the lipid moiety of representatives of the three species revealed that the N-terminus of the lipid moiety is acylated with a long-chain fatty acid (C17) in *S. aureus* and *S. epidermidis*, while it is only acetylated with a short-chain fatty acid (C2) in *S. carnosus*. Consequently, *S. carnosus* triggered a much higher immune response than *S. aureus* and *S. epidermidis*. The difference in immune response is comparable to the immune response elicited by tri- and di-acylated lipopeptides, respectively. The short-chain fatty acid (C2) structure in *S. carnosus* is apparently so small that it behaves similar as a diacylated lipopeptide. This result is in so far important as it also points in favor of the assumption that the Lpp lipid structure is involved in tolerance vs. non-tolerance by our immune system – or otherwise said, through their lipid structures bacterial species may establish their ecological niche as commensals. As *S. carnosus* induces such a fulminant immune reaction it is not surprising that it never has been observed as human commensal.

Although it is well accepted that the di- and tri-acylation of the lipid structure of Lpp modulate our immune system differently, there is no information of how the length of O-acylated fatty acids or the Lyso-Lpp modulate our immune system; this area still requires additional elucidation.

## How Firmly Are Lpp Anchored and How Are They Released

There is evidence in Gram-negative bacteria that amino acids next to the cysteine residue (+1) in mature Lpp have an impact on the strength of the anchoring of Lpp in the membrane. In *E. coli*, the amino acid at position +2 determines the localization of Lpp either in the inner or outer membrane (Gennity et al., 1992). In Gram-negative bacteria, the ABC transporter LolCDE complex translocates outer membrane-specific lipoproteins (Lpp) from the inner membrane to the outer membrane. However, Lpp possessing an aspartate (Asp) at position +2 are not translocated because this residue functions as a LolCDE avoidance signal (Fukazawa et al., 2010; Narita and Tokuda, 2011). Since Gram+ bacteria lack the typical outer membrane and the Lol system, Lpp are anchored only in the cytoplasmic membrane by integrating their lipid portion. Investigating whether a similar effect could be observed also in Gram+ bacteria revealed that in *S. aureus* Asp at position +2 plays also a role in withholding Lpp to the cytoplasmic membrane. A replacement of Asp+2 by Ser caused a higher release of the corresponding Lpp (Kumari et al., 2017). It is assumed that Asp in position +2 and adjacent amino acids contribute in tightening the anchoring of Lpp by interaction of the negatively charged Asp with the positively charged Lys-PG (Kumari et al., 2017). In mycobacteria, some Lpp may also be anchored in the mycolic layer which were recently considered as the outer membrane (Bansal-Mutalik and Nikaido, 2014; Becker and Sander, 2016). Even though no Lol homologs could be identified yet, protein-protein interaction studies suggested that two Lpp, LppK and LppI, could be transported by the lipid transport protein LprG (Touchette et al., 2017). Furthermore, the study showed that LprG interacts with the mycolyl-transferase Ag85A, and that a deletion of either lprG or ag85A gene affects growth and mycolylation (Touchette et al., 2017).

Lipoproteins are not exclusively anchored in the bacterial membrane, to a certain extent they are also released in their surrounding environment. The precise mechanism how Lpp are released into the environment is still under investigation. Recently, it was demonstrated that they are released as part of bacterial membrane vesicles (BMVs). One of the first vesicle-like blebbing structure in Gram+ bacteria was reported on the surface of *Bacillus* (Dorward and Garon, 1990). Now we know that such membrane vesicles (MVs) are produced by all domains of life. Bacterial membrane vesicles (BMVs) have been studied in *Clostridium perfringens*, *Listeria monocytogenes*, *Mycobacterium tuberculosis*, *Streptococcus pyogenes*, *Staphylococcus aureus* or *Streptomyces* spp. BMVs carry quite heterogenous containing membranous components, nucleic acids, toxins, enzymes and Lpp which contribute to the manifold activities of such BMVs in microbial physiology and pathogenesis as reviewed by Brown et al., 2015.

*Streptococcus pyogenes* releases Lpp after treatment of the cells with sublethal concentrations of penicillin in form of Lpp-rich BMVs (Biagini et al., 2015). Penicillin weakens the bacterial cell wall. Interestingly, lipid and proteomic analysis of the vesicles revealed that they were enriched of phosphatidylglycerol and almost exclusively composed of Lpp. This example shows that under antibiotic treatment of a patient BMVs can be formed and may influence the course of infection.

After invasion of macrophages *M. tuberculosis* also forms BMVs loaded with lipoglycans and Lpp (Athman et al., 2015). BMVs are here regarded as the primary means how *M. tuberculosis* exports lipoglycans and Lpp to impair effector functions of infected macrophages and circulate bacterial components beyond the site of infection, in order to regulate immune responses or iron acquisition. In the regulation of BMV production and vesiculogenesis there are various genes involved, including *virR*, the Pst/SenX3-RegX3 signal transduction system and the ESX-5 protein secretion system (White et al., 2018). One of the *M. tuberculosis* extracellular vesicle-associated Lpp (LpqH) can be used as a potential biomarker to distinguish *M. paratuberculosis* infection or vaccination from infection due to *M. tuberculosis* (Palacios et al., 2019).

In *S. aureus* the release of Lpp is boosted by the production of phenol-soluble modulin (PSM) peptides, which act as membrane detergents and surfactants (Hanzelmann et al., 2016). They damage both bacterial and host membranes. The damage of the bacterial cytoplasmic membrane also causes the release of Lpp which enhances the TLR2-mediated innate immune response. Thus, PSM surfactants in *S. aureus*, mainly the alpha PSMs, exert a similar effect as penicillin in *S. pyogenes*. Whenever, the membrane is damaged directly by PSMs or indirectly e.g., by ß-lactam antibiotics, one can expect a release of membrane-bound proteins like Lpp. However, it was also found that PSMs promote the release of BMVs from the cytoplasmic membrane which contains high amounts of Lpp (Schlatterer et al., 2018). There is evidence that the bacterial turgor is the driving force for vesicle budding under hypotonic osmotic conditions. The underlying mechanism of the release of Lpp and BMVs is subject of ongoing research. Many questions arise: for instance, if Lpp are primarily imbedded within the BMV membrane or anchored with their lipid part in the membrane, how can they induce TRL2 response. Overall, the influence of Gram+ BMVs on health and disease has by far not yet been fully clarified (Liu et al., 2018) leaving room for pertinent and exciting research.

## Application of Lpp as Adjuvants and Vaccine

Lipoproteins play a role as adjuvant and as antigens for vaccine development. Lpp and their synthetic analogs are strong immune modulators of the early host responses after infection. Synthetic lipopeptides are strong adjuvants for the adaptive immune system. Particularly the water-soluble lipohexapeptide Pam3Cys-Ser-(Lys)_4_ (PCSL) triggers a high IL-8 induction via TLR2 and constitutes a potent immune adjuvant and induces virus specific CD8(+) T cells in mice when covalently coupled to a synthetic peptide (Schild et al., 1991; Spohn et al., 2004).

Freund’s complete adjuvant (FCS) can be replaced by some specific lipopeptides (e.g., PCSL) with the antigen specific IgM response after 7 days, and an ensuing IgG response after 14 days (Reitermann et al., 1989). Among other antigens, inactivated coronavirus strain 800 was also tested (Hofmann et al., 1996). Only after the first booster immunization did the content of specific IgY antibodies in the sera increase significantly, especially in the animals first immunized using PCSL as adjuvants (Hofmann et al., 1996). Although this work dates back almost 25 years, the positive effect of PCSL on the formation of corona virus specific antibodies shows how seminal this study is still today. These results illustrate that synthetic lipopeptides could be extremely valuable for preventive or therapeutic use. Indeed, recently a new water-soluble synthetic Pam3Cys-GDPKHPKSF named XS15 has been designed. Tolerability and immune responses of XS15 together with an antigen were closely monitored (in a single human volunteer). A granuloma formed at the injection site in which activated and functional CD4(+) and CD8(+) effector memory T cells had accumulated and were still detectable after one year. The lipopeptide XS15 was considered a promising adjuvant for tumor peptide vaccination (Rammensee et al., 2019).

Since the protein portion of many Lpp spans the cell wall and is therefore accessible for antibodies, it is obvious to use them for vaccine development. For the therapy of *Enterococcus* infections two metal binding Lpp were tested as vaccine candidates, namely the manganese ABC transporter substrate-binding Lpp (PsaAfm), and the zinc ABC transporter substrate-binding Lpp (AdcAfm) (Romero-Saavedra et al., 2015). The two antigens elicited specific, opsonic and protective antibodies, with an extensive cross-reactivity and serotype-independent coverage. The use of recombinant Lpp with built-in immunostimulating properties for novel subunit vaccine development was reviewed recently (Leng et al., 2015). Promising results were obtained with a dengue subunit vaccine, with a novel subunit vaccine against *Clostridium difficile*-associated diseases and with HPV-based immunotherapeutic vaccines. Against *S. aureus* a cocktail of five conserved antigens was tested containing two Lpp, i.e., the ferric hydroxamate-binding Lpp FhuD2 and the putative Lpp named conserved staphylococcal antigen 1A (Csa1A) as surface-exposed antigens (Bagnoli et al., 2015). These data demonstrated that the rational selection of mixtures of conserved antigens combined with Th1/Th17 adjuvants may lead to promising vaccine formulations against *S. aureus*. Remarkably, a breakthrough was achieved when these cocktail antigens were used together with a TLR7 agonist adsorbed to alum as adjuvants (Bagnoli et al., 2015). Altogether, the potential for the future use of Lpp in vaccination is still by far not fully appreciated even though these exciting molecules provide an extremely promising basis with the respect in vaccine development.

## Conclusion

A total of 50 years have passed since first discovery of the Braun’s Lpp in *E. coli*. In the meantime, we know much more about the biosynthesis of Lpp and their functions. We know that Lpp occur in all Gram+ bacteria. The tasks they perform in the respective species are extremely diverse. Some of the described Lpp are absolutely essential for the life of the bacteria. Many Lpp contribute to fitness and become essential under certain nutrient limiting conditions. Lpp consist of the lipid and the protein part. Both have an important but completely different function and activity. While the protein part can have the most diverse binding and enzymatic activities, the lipid part together with a small peptide residue modulates the immune system. Yet, despite this enormous insight in structure and function of Lpp, there are still large gaps in our knowledge, for example, approximately 30% of Lpp in Gram+ bacteria are still uncharacterized with respect to function. Moreover, the ascertainment that the lipid structure of Gram+ bacteria in particular is so variable has only come to light in recent years, a fact largely owed to the methodical challenges of lipid structure analyses. The discovery of new lipid variants, their activity on the immune system, as well as the search for the modifying enzymes will thus be important aims for research during the upcoming years, and it is strongly expected that ensuing findings will allow for opening novel avenues in the use of Lpp in diagnostic, preventive or therapeutic medicine.

## In Honor of Volkmar Braun the Discoverer of Lpp

This review article is also in honor of Volkmar Braun at the University Tübingen, who discovered bacterial lipoproteins (Lpp) almost 50 years ago. His discovery of the Lpp was based on accurate scientific work, careful analysis of the results and drawing the right conclusions. He himself describes the first observations which finally led to the discovery of “Braun’s lipoprotein” as follows:

“In attempts to isolate membrane proteins from *E. coli*, isolated cell walls were treated with trypsin which caused the fastest decrease of the optical density among all tested proteases. Murein (peptidoglycan) from trypsin-treated cell walls, isolated with hot 4% sodium dodecyl sulfate was free of protein but contained additional lysine and arginine residues which presumably were left-overs from trypsin cleavage (Braun and Rehn, 1969).

The second key observation was the peptide Arg-Lys-Dpm in an acid hydrolysate which showed the attachment site of the protein at Dpm (diaminopimelate) of the murein peptide side chain (Braun and Bosch, 1972). Six amino acids commonly found in proteins were lacking in the protein. This data pointed to a protein of a defined size and composition covalently attached to the murein via the Arg-Lys bond.

Another highlight was the determination of the unique lipid structure covalently linked to the protein (Hantke and Braun, 1973), which is now predicted to be contained in approximately 2.7% of bacterial membrane proteins. Since nearly one million Lpp molecules are evenly distributed over the murein net, one can deduce a model of the Lpp organization in the outer membrane. Many functions were assigned to Lpp, as this review lucidly demonstrates, but most of them are not even touched.

Before the discovery of the major *E. coli* Lpp, the term lipoprotein was used for undefined mixtures of aggregated proteins with all sorts of lipids. No Lpp and even no membrane protein had been sequenced. The Lpp was also the first protein whose heptad repeat sequence fulfilled the prediction of Francis Crick in 1953 of coiled-coil structures.”

## Author Contributions

M-TN and FG wrote the manuscript. MM, SN, and MH contributed to revise the manuscript. All authors contributed to the article and approved the submitted version.

## Conflict of Interest

The authors declare that the research was conducted in the absence of any commercial or financial relationships that could be construed as a potential conflict of interest.

## Abbreviations

Lpp: Lipoprotein(s)
*lpl*: lipoprotein-like lipoprotein genes
Lps: lipopolysaccharides
*lgt*: diacylglyceryl transferase enzyme encoding gene
Lsp: signal peptidase II encoding gene
PGN: peptidoglycan
TLR2: Toll-like receptor 2
*B.*: *Bacillus*
C.: Clostridium
E.: Enterococcus
G.: *Geobacillus*
L.: *Listeria*
M.: Mycobacterium
O.: Oceanobacillus
*S.*: *Staphylococcus* or *Streptococcus*.
