# Supplementary material for: Lipoproteins in Gram-Positive Bacteria: Abundance, Function, Fitness
Source: Front Microbiol. 2020 Sep 18;11:582582. doi: 10.3389/fmicb.2020.582582 (PMC7530257; doi:10.3389/fmicb.2020.582582)
Supplement: Supplementary file 1 [file Table_1.docx]

**Supplementary tables**

**S1. Lpp of *Bacillus subtilis***

| **No** | **Locus tag** | **Function/Annotation** | **SPII (aa)** | **Lipobox** | **Mass (KDa)** |
| --- | --- | --- | --- | --- | --- |
|  |  | **Fe transportation** |  |  |  |
| 1 | NP_388265 | petrobactin iron-siderophore ABC transporter | 19 | ISA | 317 |
| 2 | NP_388633 | iron-dicitrate ABC transporter | 24 | LLA | 315 |
| 3 | NP_388725 | xenosiderophore schizokinen (dihydroxamate) transporter binding | 20 | LSA | 325 |
| 4 | NP_391213 | ferrichrome ABC transporter | 23 | LAA | 315 |
| 5 | NP_391840 | desferrioxamine-and ferrichrome-binding transporter lipoprotein | 20 | VSA | 321 |
|  |  | **Other cation transportation** |  |  |  |
| 6 | NP_388167 | Zn(II)-binding lipoprotein | 20 | VAA | 319 |
| 7 | NP_390560 | lipoprotein buffering protein for Zn2+ transport | 22 | LAG | 251 |
| 8 | NP_390815 | sulfur-containing amino acid ABC transporter | 20 | LSA | 270 |
| 9 | NP_390955 | manganese ABC transporter | 18 | LTG | 306 |
| 10 | NP_391218 | molybdate-binding lipoprotein | 20 | VAG | 260 |
|  |  | **Anion transportation** |  |  |  |
| 11 | NP_388454 | putative phosphate-starvation lipoprotein | 16 | LAG | 236 |
| 12 | NP_388764 | aliphatic sulfonate ABC transporter | 17 | LAG | 332 |
| 13 | NP_390937 | putative ABC anion transporter component | 23 | LAA | 334 |
|  |  | **Amino acid and peptide transportation** |  |  |  |
| 14 | NP_388220 | amino acid ABC transporter (binding lipoprotein subunit) | 24 | LAA | 287 |
| 15 | NP_388243 | cystine ABC transporter | 19 | LAA | 268 |
| 16 | AAA62358 | AppA | 23 | LSA | 543 |
| 17 | NP_389025 | oligopeptide ABC transporter | 20 | LSA | 545 |
| 18 | NP_390278 | high affinity arginine ABC transporter | 19 | LTA | 255 |
| 19 | NP_390621 | glutamine ABC transporter | 20 | LAA | 273 |
| 20 | NP_391251 | choline ABC transporter | 22 | LSG | 306 |
| 21 | NP_388182 | L-proline betaine and betonicine ABC transporter | 20 | LAA | 293 |
| 22 | NP_391198 | putative lipoprotein binding vitamin B12 | 20 | LAG | 314 |
| 23 | NP_391836 | lipocalin-like lipoprotein, YxeF family protein | 23 | VSG | 144 |
|  |  | **Sugar transportation** |  |  |  |
| 24 | NP_388591 | lipoprotein transporter binding protein for alpha-galacturonides | 21 | IAG | 502 |
| 25 | NP_390753 | sugar-binding lipoprotein | 21 | IAG | 433 |
| 26 | NP_391140 | fructose amino acid-binding lipoprotein | 20 | IAG | 422 |
| 27 | NP_391296 | polygalactose / cyclodextrin-binding lipoprotein | 22 | LAA | 421 |
| 28 | NP_391341 | maltose/maltodextrin-binding lipoprotein | 22 | LAA | 417 |
| 29 | NP_391477 | ribose ABC transporter (ribose-binding lipoprotein) | 18 | LTA | 305 |
|  |  | **Unknown transportation** |  |  |  |
| 30 | NP_388539 | putative lipoprotein (YecC) | 19 | LVS | 331 |
| 31 | NP_391829 | putative ABC transporter (binding lipoprotein) | 20 | LSA | 264 |
|  |  | **Cell wall** |  |  |  |
| 32 | NP_389345 | small peptidoglycan-associated lipoprotein | 18 | LSG | 124 |
| 33 | NP_390300 | putative lipoprotein YqiH | 16 | LSG | 97 |
| 34 | NP_391444 | membrane bound lipoprotein LytA | 16 | LSG | 102 |
| 35 | NP_391759 | putative exported polysaccharide deacetylase, lipoprotein | 19 | LAA | 279 |
| 36 | NP_388217 | putative Zn(2+)-dependent division lipoprotein (schizosome) | 19 | LSA | 194 |
| 37 | NP_388295 | penicillin-binding lipoprotein 3; transpeptidase | 20 | LIG | 668 |
|  |  | **Germination** |  |  |  |
| 38 | NP_388036 | lipoprotein factor mediating clustering of germination proteins | 19 | VTA | 185 |
| 39 | NP_388659 | putative spore germination protein | 20 | LTG | 384 |
| 40 | NP_389363 | putative germination lipoprotein | 16 | LSA | 209 |
| 41 | NP_389660 | putative spore germination lipoprotein | 24 | VTG | 404 |
| 42 | NP_390716 | germination (cortex hydrolysis) and sporulation (stage II, multiple polar septa) lytic enzyme | 22 | LSG | 366 |
| 43 | NP_391112 | putative sporulation-related lipoprotein | 17 | LSG | 210 |
| 44 | NP_391174 | spore lipoprotein | 19 | IAG | 145 |
| 45 | NP_391187 | component of the germination receptor GerA | 17 | LSG | 373 |
| 46 | AIY95448 | sporulation protein | 20 | LSA | 335 |
|  |  | **Enzymes and foldases** |  |  |  |
| 47 | NP_388876 | molecular chaperone lipoprotein | 19 | LSA | 292 |
| 48 | NP_391407 | cytochrome c551 | 20 | LAA | 112 |
| 49 | NP_389644 | DNA nuclease, lipoprotein | 19 | LAA | 211 |
| 50 | NP_388545 | CamS family sex pheromone protein | 18 | LSA | 396 |
| 51 | NP_389984 | putative hydrolase lipoprotein; phage SPbeta | 21 | LAA | 203 |
| 52 | NP_390057 | putative exported lipase/acylhydrolase | 18 | LTA | 255 |
| 53 | NP_390044 | calcium-dependent DNA nuclease, lipoprotein; phage SPbeta | 19 | LAA | 296 |
| 54 | NP_389372 | cytochrome caa3 oxidase | 20 | LSG | 356 |
| 55 | NP_389822 | superoxide dismutase (exported lipoprotein) SOD | 17 | VAG | 196 |
| 56 | NP_391984 | Sec-independent factor for membrane protein insertion (YidC/SpoIIIJ) | 22 | LAG | 261 |
| 57 | NP_389012 | positive regulator of comK | 17 | LSG | 317 |
|  |  | **Unknown function** |  |  |  |
| 58 | NP_388380 | ICEBs1 mobile element: putative lipoprotein of unknown function | 18 | LTA | 216 |
| 59 | NP_388403 | putative lipoprotein | 22 | LAG | 219 |
| 60 | NP_389100 | putative lipoprotein | 19 | LSA | 213 |
| 61 | NP_389762 | hypothetical protein | 20 | LAG | 120 |
| 62 | NP_390767 | putative lipoprotein | 17 | LTG | 210 |
| 63 | NP_388344 | Putative Lpp | 23 | LSA | 338 |

**S2. Lpp of *Bacillus cereus***

| **No** | **Locus tag** | **Function/Annotation** | **SPII (aa)** | **Lipobox** | **Mass (KDa)** |
| --- | --- | --- | --- | --- | --- |
|  |  | **Fe transportation** |  |  |  |
| 1 | AAP07634 | Iron(III) dicitrate-binding protein | 24 | LSA | 322 |
| 2 | AAP09174 | iron-siderophore ABC transporter substrate-binding | 21 | IAG | 320 |
| 3 | AAP10401 | Ferrichrome binding protein ABC transporter | 19 | LAA | 302 |
| 4 | AAP10664 | iron (III)dicitrate-binding protein ABC transporter | 20 | LAA | 321 |
| 5 | AAP11276 | Ferrichrome binding protein ABC transporter | 20 | LIG | 315 |
| 6 | AAP11329 | Ferrichrome binding protein ABC transporter | 20 | IVG | 314 |
| 7 | QCX96363 | heme ABC transporter substrate-binding protein IsdE | 21 | IAG | 293 |
| 8 | AAP11975 | Ferric anguibactin-binding protein | 22 | LVA | 338 |
|  |  | **Amino acid and peptide transportation** |  |  |  |
| 9 | AAP07284 | peptide ABC transporter substrate-binding protein | 21 | LTA | 546 |
| 10 | AAP07285 | peptide ABC transporter substrate-binding protein | 21 | LTG | 546 |
| 11 | AAP07310 | ABC transporter oligo-binding protein (oppA) | 24 | LSA | 575 |
| 12 | QCX92408 | MetQ/NlpA family ABC transporter substrate-binding protein | 20 | LSG | 280 |
| 13 | AAP07466 | ABC transporter, Hydroxymethylpyrimidine-binding protein | 19 | VAG | 332 |
| 14 | AAP07657 | glutamine ABC transporter substrate-binding protein GlnH | 23 | VAG | 276 |
| 15 | AAP07859 | amino acid ABC transporter, cystine-binding protein | 20 | VAG | 265 |
| 16 | AAP08165 | peptide ABC transporter, oppA | 21 | LSA | 551 |
| 17 | AAP08272 | spermidine/putrescine ABC transporter substrate-binding protein PotD | 23 | LAG | 349 |
| 18 | AAP08995 | peptide ABC transporter, oppA | 21 | VAG | 553 |
| 19 | AAP09798 | peptide ABC transporter, oppA | 21 | LVG | 540 |
| 20 | AAP10517 | Oligopeptide-binding protein oppA | 17 | LTA | 556 |
| 21 | QCX95433 | peptide ABC transporter | 25 | LTA | 571 |
| 22 | AAP10519 | peptide ABC transporter substrate, oppA | 25 | LTA | 567 |
| 23 | AAP11856 | MetQ/NlpA family ABC transporter | 19 | LAA | 268 |
| 24 | AAP11857 | MetQ/NlpA family ABC transporter substrate | 19 | LAA | 270 |
| 25 | AAP08171 | Oligopeptide-binding protein oppA | 21 | LGA | 548 |
| 26 | QCX92301 | peptide ABC transporter substrate-binding protein | 19 | SVA | 536 |
| 27 | QCX93731 | peptide ABC transporter substrate-binding protein | 17 | LTA | 542 |
| 28 | QCX95432 | peptide ABC transporter substrate-binding protein | 25 | LTA | 564 |
|  |  | **Sugar transportation** |  |  |  |
| 29 | AAP07759 | sugar ABC transporter, Methylthioribose-binding protein | 18 | LAA | 370 |
| 30 | AAP09908 | ABC transporter sugar-binding (LsrB) | 20 | LIA | 247 |
|  |  | **Unknown transportation** |  |  |  |
| 31 | AAP08661 | ABC transporter substrate-binding protein | 23 | VTG | 341 |
|  |  | **Cell wall** |  |  |  |
| 32 | AAP09159 | Mec A protein (penicillin-binding protein 3) | 18 | LVG | 661 |
| 33 | AAP09430 | Cell elongation specific D,D- transpeptidase | 18 | LVG | 655 |
| 34 | AAP10147 | Succinoglycan biosynthesis protein | 18 | VTG | 445 |
| 35 | AAP10248 | Capsule biosynthesis protein CapA | 24 | LTA | 411 |
| 36 | AAP07401 | polysaccharide deacetylase family protein | 17 | LAG | 360 |
|  |  | **Germination** |  |  |  |
| 37 | AAP07249 | germination protein GerD | 19 | LAA | 205 |
| 38 | AAP07650 | spore germination protein GerKC | 20 | LSG | 374 |
| 39 | QCX94539 | Ger(x)C family spore germination protein | 19 | ISG | 386 |
| 40 | QCX04640 | YhcN/YlaJ family sporulation lipoprotein | 16 | ITG | 198 |
| 41 | AAP10041 | Spore germination protein BC | 16 | LIG | 359 |
| 42 | AAP10054 | Spore germination protein BC | 25 | FLT | 397 |
| 43 | AAP10509 | spore germination protein GerSC | 19 | LTG | 378 |
| 44 | AAP11406 | spore germination protein GerM | 21 | LTG | 349 |
| 45 | AAP11638 | spore germination protein IC | 23 | LIG | 361 |
|  |  | **Enzymes and foldases** |  |  |  |
| 46 | Q515V9 | membrane protein insertase YidC 1 | 22 | LSG | 260 |
| 47 | Q814F4 | membrane protein insertase YidC 2 | 20 | ATG | 258 |
| 48 | AAP08804 | Copper resistant protein A - multicopper oxidase family protein | 19 | IAA | 546 |
| 49 | AAP07382 | CamS family sex pheromone protein | 17 | VSG | 398 |
| 50 | QCX92858 | ester cyclase | 21 | LVA | 179 |
| 51 | Q81GY5 | peptidylprolyl isomerase PrsA 1 | 18 | LSA | 286 |
| 52 | Q81GN0 | peptidylprolyl isomerase PrsA 2 | 20 | LSA | 285 |
| 53 | AAP09168 | SCO family protein (Cytochrome c oxidase Cu(A)center assembly protein) | 21 | LAG | 195 |
| 54 | Q81DT1 | foldase protein PrsA 3, peptidylprolyl isomerase | 21 | LSA | 283 |
| 55 | PRSA4_BACCR | foldase protein PrsA 4 | 21 | LSA | 280 |
| 56 | AAP11258 | Lipase | 19 | LVG | 294 |
| 57 | AAP11421 | Acid phosphatase, 5'-nucleotidase, lipoprotein e(P4) family | 22 | LVA | 275 |
|  |  | **Unknown function** |  |  |  |
| 58 | AAP07274 | hypothetical protein BC_0205 | 18 | LAG | 167 |
| 59 | AAP07281 | hypothetical protein | 19 | LTG | 155 |
| 60 | AAP07399 | DUF4352 domain-containing protein | 20 | LSA | 163 |
| 61 | AAP07402 | hypothetical protein | 18 | IVG | 235 |
| 62 | QCX92597 | hypothetical protein EJ379_02885 | 20 | VVG | 167 |
| 63 | AAP07599 | DUF4871 domain-containing protein | 16 | ITG | 174 |
| 64 | QCX92632 | hypothetical protein EJ379_03080 | 21 | LSG | 172 |
| 65 | AAP07772 | hypothetical protein | 18 | LAG | 170 |
| 66 | AAP07984 | hypothetical protein BC_0997 | 19 | LIG | 151 |
| 67 | AAP08017 | hypothetical protein BC_1030 | 18 | LSG | 219 |
| 68 | AAP08282 | hypothetical protein, surface antigen | 16 | LVG | 324 |
| 69 | AAP08770 | hypothetical protein BC_1796 | 18 | LSA | 126 |
| 70 | AAP08906 | DUF4352 domain-containing protein | 20 | LSA | 179 |
| 71 | AAP08946 | YkyA family protein | 19 | LTG | 218 |
| 72 | AAP09051 | Hypothetical protein BC-2082 (DUF3952 domain-containing protein) | 22 | LSG | 266 |
| 73 | AAP09059 | Hypothetical protein BC_2090 (DUF3952 domain-containing protein) | 21 | LSA | 273 |
| 74 | AAP09177 | hypothetical protein BC-2211 | 16 | LVG | 162 |
| 75 | QCX94454 | hypothetical protein EJ379_13025 | 20 | VSA | 223 |
| 76 | AAP09560 | hypothetical protein BC_2601 | 18 | LVG | 250 |
| 77 | AAP09724 | hypothetical protein | 20 | LTG | 163 |
| 78 | AAP09835 | Hypothetical protein BC_2886 (YjgB family protein) | 23 | LAA | 223 |
| 79 | AAP09844 | hypothetical protein BC_2895 | 17 | LSG | 120 |
| 80 | AAP09855 | Hypothetical protein BC_2906 (DUF4825 domain-containing protein) | 20 | IAA | 197 |
| 81 | AAP10002 | hypothetical protein BC_3055 | 19 | LSG | 326 |
| 82 | AAP10141 | hypothetical protein | 17 | LTA | 156 |
| 83 | AAP10156 | hypothetical protein BC_3214 | 18 | VLG | 182 |
| 84 | AAP10199 | hypothetical protein | 21 | ITG | 153 |
| 85 | AAP10291 | hypothetical protein BC_3351 | 17 | LSG | 125 |
| 86 | AAP10795 | Hyphothetical protein BC_3874 DUF1311 domain-containing protein | 21 | ITG | 393 |
| 87 | AAP10916 | YkyA family protein | 20 | LAG | 220 |
| 88 | AAP10938 | Hypothetical protein BC_4019 DUF3997 domain-containing protein | 15 | LTG | 143 |
| 89 | QCX95920 | IG hypothetical EJ379_20910 | 18 | LAG | 247 |
| 90 | AAP11286 | hypothetical protein BC_4373 | 20 | LAA | 232 |
| 91 | AAP11332 | hypothetical protein BC_4419 | 20 | LAA | 214 |
| 92 | AAP11522 | Hypothetical protein BC_4615, YobA family protein | 20 | LSA | 121 |
| 93 | APP11586 | hypothetical protein BC_4679 | 20 | LIG | 144 |
| 94 | AAP11705 | hypothetical protein BC_4802 | 20 | LSG | 192 |
| 95 | AAP11967 | Hypothetical protein BC_5098 (DUF4822 domain-containing protein) | 21 | LTG | 321 |
| 96 | AAP12034 | Hypothetical protein BC_5169 (DUF4362 domain-containing protein) | 19 | LVA | 143 |

**S3. Lpp of *Bacillus licheniformis***

| **No** | **Locus tag** | **Function/Annotation** | **SPII (aa)** | **Lipobox** | **Mass (KDa)** |
| --- | --- | --- | --- | --- | --- |
|  |  | **Fe transportation** |  |  |  |
| 1 | AAU25696 | Periplasmic binding protein YxeB | 20 | VSA | 319 |
| 2 | AAU24171 | ferrichrome ABC transporter | 23 | LTA | 315 |
| 3 | AAU24348 | Periplasmic iron-binding protein | 22 | LSG | 318 |
| 4 | AAU24963 | putative iron transport system substrate-binding protein YvrC | 20 | LSG | 316 |
| 5 | AAU25109 | Periplasmic binding protein,putative iron transporter | 19 | LAA | 315 |
|  |  | **Anion transportation** |  |  |  |
| 6 | AAU22505 | aliphatic sulfonate ABC transporter | 17 | LAG | 329 |
| 7 | AAU24699 | putative sulfonate transport system substrate-binding protein YtlA | 20 | LAS | 330 |
|  |  | **Amino acid and peptide transportation** |  |  |  |
| 8 | AAU22788 | oligopeptide ABC transporter | 23 | LTA | 547 |
| 9 | AAU22794 | oligopeptide ABC transporter | 20 | LSA | 541 |
| 10 | AAU24044 | oligopeptide ABC transporter | 20 | LAS | 509 |
| 11 | AAU24141 | glycine betaine ABC transporter | 20 | LAA | 294 |
| 12 | AAU25103 | glycine betaine/carnitine/choline ABC transporter | 22 | ISG | 305 |
| 13 | AAU23645 | possible C4-dicarboxylate binding protein | 20 | LTG | 333 |
|  |  | **Sugar transportation** |  |  |  |
| 14 | AAU39220 | putative D-xylose ATP transporter permease XylF | 24 | VAG | 354 |
| 15 | AAU22935 | lipoprotein, carbohydrate ABC transporter substrate-binding protein | 23 | LIG | 507 |
| 16 | AAU22042 | ribose ABC transporter (ribose-binding protein) | 19 | VTA | 359 |
| 17 | AAU22248 | maltodextrin transport system substrate-binding protein MdxE | 22 | LAA | 417 |
| 18 | AAU22701 | multiple sugar-binding protein MsmE | 20 | LAG | 417 |
| 19 | AAU24531 | sugar-binding protein | 21 | LSG | 434 |
| 20 | AAU25288 | ribose ABC transporter | 18 | LSA | 304 |
|  |  | **Unknown transportation** |  |  |  |
| 21 | AAU21998 | ABC transport system substrate-binding protein | 21 | LSG | 360 |
| 22 | AAU22539 | ABC transport system protein | 17 | IAG | 272 |
| 23 | AAU24074 | Taxi family TRAP transporter solute-binding subunit | 22 | LAA | 329 |
| 24 | AAU24913 | ABC transport system substrate-binding protein | 19 | LAA | 276 |
| 25 | AAU24933 | ABC transport system substrate-binding protein | 20 | LAA | 325 |
|  |  | **Cell wall** |  |  |  |
| 26 | AAU23218 | small peptidoglycan-associated lipoprotein | 18 | LSG | 125 |
| 27 | AAU24105 | YqiH | 17 | LAG | 106 |
|  |  | **Germination** |  |  |  |
| 28 | AAU21802 | spore germination protein D (GerD) | 19 | VTA | 193 |
| 29 | AAU22151 | conserved membrane protein YdcC | 20 | LSA | 335 |
| 30 | AAU23700 | spore germination B3 GerAC like, C-terminal | 21 | LTG | 398 |
| 31 | AAU24416 | spore cortex protein | 20 | LSA | 211 |
| 32 | AAU24492 | spore germination protein GerM | 22 | LSG | 369 |
| 33 | AAU24880 | conserved protein YutC | 17 | LAG | 207 |
| 34 | AAU24948 | spore germination protein A3 precursor | 18 | LTG | 373 |
|  |  | **Enzymes and foldases** |  |  |  |
| 35 | AAU22635 | molecular chaperone PrsA | 19 | LSA | 286 |
| 36 | AAU25214 | cytochrome c551 | 18 | LAA | 108 |
| 37 | AAU21907 | putative Proteinase inhibititor I4, serpin | 19 | LAG | 404 |
| 38 | AAU22761 | intracellular proteinase inhibitor Ipi | 15 | VSG | 150 |
| 39 | AAU22780 | transcriptional activator protein Med | 17 | LSG | 317 |
| 40 | AAU23835 | conserved protein Ypm, SGNH/GDSL hydrolase family protein | 18 | LSA | 250 |
| 41 | AAU23836 | conserved hypothetical YpmQ, cytochrome c oxidase assembly protein | 19 | LSS | 193 |
| 42 | AAU24274 | putative lipoprotein, SGNH/GDSL hydrolase family protein | 17 | LSG | 243 |
| 43 | AAU23245 | cytochrome caa3 oxidase | 20 | LAG | 355 |
| 44 | AAU43185 | membrane protein translocase SpoIIIJ | 20 | LLA | 265 |
|  |  | **Unknown function** |  |  |  |
| 45 | AAU22065 | Periplasmic binding protein | 19 | VAA | 315 |
| 46 | AAU22298 | conserved protein YerB | 20 | LAS | 329 |
| 47 | AAU22303 | YerH | 18 | LSA | 396 |
| 48 | AAU22968 | hypothetical protein BL03732 | 21 | LAG | 221 |
| 49 | AAU22981 | hypothetical protein BL05123 | 19 | LAA | 173 |
| 50 | AAU23115 | hypothetical protein BL00544 | 18 | LTA | 184 |
| 51 | AAU23236 | hypothetical protein BL02997 | 17 | LSA | 204 |
| 52 | AAU23544 | conserved hypothetical protein | 20 | LSG | 118 |
| 53 | AAU24664 | conserved protein YteS | 19 | LTS | 164 |
| 54 | AAU24677 | putative lipoprotein | 22 | LAA | 274 |
| 55 | AAU24706 | conserved protein YtkA | 18 | LSA | 147 |
| 56 | AAU25715 | putative extracellular solute-binding protein, family 1 CycB | 22 | LTA | 422 |
| 57 | AAU25253 | membrane bound lipoprotein | 17 | LSA | 106 |

**S4. Lpp of *G. kaustophilus***

| **No** | **Locus tag** | **Function/Annotation** | **SPII (aa)** | **Lipobox** | **Mass (KDa)** |
| --- | --- | --- | --- | --- | --- |
|  |  | **Fe transportation** |  |  |  |
| 1 | BAD74478 | ferrichrome ABC transporter | 18 | LSA | 308 |
| 2 | BAD75742 | iron(III) dicitrate ABC transporter | 21 | LAG | 322 |
| 3 | BAD76551 | ferric ion ABC transporter | 22 | LVG | 319 |
|  |  | **Anion transportation** |  |  |  |
| 4 | BAD76748 | phosphate ABC transporter | 21 | LAG | 296 |
| 5 | BAD77140 | nitrate/sulfonate/taurine/bicarbonate ABC transporter | 19 | LSA | 334 |
|  |  | **Amino acid and peptide transportation** |  |  |  |
| 6 | BAD74600 | hypothetical protein GK0315, glycine/betaine ABC transporter substrate binding protein | 19 | LAA | 299 |
| 7 | BAD75949 | branched-chain amino acid ABC transporter | 22 | LAA | 404 |
| 8 | BAD76259 | C4-dicarboxylate transport system | 20 | LSG | 342 |
| 9 | BAD76262 | glutamine ABC transporter | 25 | LTG | 281 |
| 10 | BAD77265 | branched chain amino acid ABC transporter | 22 | LAG | 398 |
| 11 | BAD77282 | amino acid ABC transporter | 19 | LAA | 283 |
| 12 | BAD77311 | branched amino acid ABC transporter | 19 | LAA | 389 |
| 13 | BAD77738 | amino acid ABC transporter | 22 | LAA | 273 |
|  |  | **Sugar transportation** |  |  |  |
| 14 | BAD75233 | ribose ABC transporter | 22 | ILA | 386 |
| 15 | BAD76166 | D-xylose ABC transporter | 22 | LSA | 363 |
|  |  | **Unknown transportation** |  |  |  |
| 16 | BAD75568 | ABC transporter (lipoprotein) | 23 | LSA | 365 |
| 17 | BAD75977 | hypothetical conserved protein, YdhK family protein, uncharacterized transporter | 21 | LSA | 188 |
| 18 | BAD76195 | ABC transporter | 19 | LSA | 359 |
| 19 | BAD76296 | ABC transporter | 19 | LVG | 368 |
| 20 | BAD76315 | ABC transporter | 21 | LSG | 337 |
| 21 | BAD76408 | ABC transporter | 22 | LAA | 439 |
| 22 | BAD77492 | ABC transporter | 21 | LSG | 432 |
|  |  | **Cell wall** |  |  |  |
| 23 | BAD75866 | D-alanyl-D-alanine carboxypeptidase | 17 | LAG | 272 |
|  |  | **Germination** |  |  |  |
| 24 | BAD75901 | spore germination protein | 17 | LSG | 383 |
| 25 | BAD75910 | spore germination protein | 20 | LSG | 402 |
|  |  | **Enzymes and foldases** |  |  |  |
| 26 | BAD76107 | stage III sporulation protein J (YidC/SpoIIIJ) | 17 | LSG | 249 |
| 27 | BAD77781 | stage III sporulation protein J (YidC/SpoIIIJ) | 20 | LAG | 254 |
| 28 | BAD77387 | cytochrome c551 | 18 | LAA | 111 |
| 29 | BAD74594 | lantibiotic biosynthesis protein | 18 | IAG | 127 |
| 30 | PRSA_GEOKA | Foldase protein PrsA | 18 | LSA | 281 |
| 31 | BAD75052 | nitrite reductase | 20 | LAA | 354 |
| 32 | BAD76055 | hypothetical conserved protein, SCO family protein | 16 | LAA | 190 |
| 33 | BAD77151 | beta-propeller fold lactonase family protein | 16 | LAG | 652 |
| 34 | BAD75367 | cytochrome c oxidase subunit II | 23 | LAG | 356 |
| 35 | BAD77217 | superoxide dismutase (Cu/Zn) | 17 | LSG | 173 |
|  |  | **Unknown function** |  |  |  |
| 36 | BAD74461 | hypothetical protein GK0176 | 20 | LVG | 173 |
| 37 | BAD74514 | hypothetical conserved protein | 20 | LAG | 318 |
| 38 | BAD74841 | hypothetical conserved protein | 20 | LVG | 230 |
| 39 | BAD74862 | hypothetical conserved protein | 18 | ISA | 157 |
| 40 | BAD75181 | hypothetical conserved protein | 18 | LSA | 250 |
| 41 | BAD75251 | hypothetical conserved protein | 18 | LAA | 199 |
| 42 | BAD75741 | hypothetical protein GK1456 | 20 | LSS | 143 |
| 43 | BAD76660 | hypothetical conserved protein | 18 | LSG | 325 |
| 44 | BAD77664 | hypothetical protein GK3379 | 15 | LSA | 146 |

**S5. Lpp of *O.iheyensis***

| **No** | **Locus tag** | **Function/Annotation** | **SPII (aa)** | **Lipobox** | **Mass (KDa)** |
| --- | --- | --- | --- | --- | --- |
|  |  | **Fe transportation** |  |  |  |
| 1 | BAC12215 | iron ABC transporter permease | 23 | LAG | 349 |
| 2 | BAC12407 | ferrichrome ABC transporter | 24 | LAA | 321 |
| 3 | BAC12498 | ferrichrome ABC transporter | 19 | LAA | 342 |
| 4 | BAC15346 | iron-siderophore binding lipoprotein | 22 | LIG | 351 |
|  |  | **Other cation transportation** |  |  |  |
| 5 | BAC13234 | metal-binding protein ZinT | 21 | ITG | 334 |
| 6 | BAC14680 | ion ABC transport- AfuA protein | 20 | LIA | 344 |
| 7 | BAC15257 | CueP family metal-binding protein | 19 | LVG | 170 |
|  |  | **Anion transportation** |  |  |  |
| 8 | BAC12558 | alkylphosphonate ABC tranporter | 19 | LAA | 320 |
| 9 | BAC12932 | phosphonate ABC transporter | 20 | LAA | 342 |
| 10 | BAC15092 | phosphate ABC transporter | 21 | LAA | 325 |
|  |  | **Amino acid and peptide transportation** |  |  |  |
| 11 | BAC12199 | C4-dicarboxylate transport system | 21 | LAG | 337 |
| 12 | BAC11983 | glycine betaine ABC transporter | 24 | LAA | 305 |
| 13 | BAC12419 | oligopeptide ABC transporter | 21 | LVG | 529 |
| 14 | BAC12643 | branched-chain amino acid ABC transporter | 21 | LTG | 399 |
| 15 | BAC12729 | oligopeptide ABC transporter | 22 | LAA | 530 |
| 16 | BAC12832 | amino acid ABC transporter | 23 | LAA | 296 |
| 17 | BAC12957 | glycine betaine ABC transporter | 18 | LAA | 288 |
| 18 | BAC12960 | glutamine ABC transporter | 21 | LAA | 272 |
| 19 | BAC14030 | amino acid ABC transporter | 19 | LSA | 268 |
| 20 | BAC14338 | MetQ/NlpA family ABC transporter substrate-binding protein | 18 | LAA | 279 |
| 21 | BAC14567 | oligopeptide ABC transporter | 23 | LAA | 613 |
| 22 | BAC14658 | C4-dicarboxylate transport | 22 | LSA | 342 |
| 23 | BAC14689 | amino acid ABC transporter | 18 | LSA | 281 |
| 24 | BAC14731 | oligopeptide ABC transporter | 19 | LTA | 524 |
| 25 | BAC14774 | C4-dicarboxylate transport | 22 | LAA | 339 |
| 26 | BAC14923 | dipeptide ABC transporter | 21 | LAA | 553 |
| 27 | BAC15027 | oligopeptide ABC transporter | 23 | LVA | 541 |
| 28 | BAC15121 | methionine ABC transporter substrate-binding protein | 19 | LAA | 287 |
| 29 | BAC15190 | tripartite tricarboxylate transporter substrate binding protein | 21 | VSA | 346 |
| 30 | BAC15205 | tripartite tricarboxylate transporter substrate binding protein | 18 | LAG | 335 |
| 31 | BAC15213 | C4-dicarboxylate transport | 19 | LAA | 329 |
| 32 | BAC15226 | tripartite tricarboxylate transporter substrate binding protein | 19 | LAA | 326 |
| 33 | BAC15283 | MetQ/NlpA family ABC transporter substrate-binding protein [ | 19 | LAA | 273 |
|  |  | **Sugar transportation** |  |  |  |
| 34 | BAC12730 | sugar ABC transporter | 19 | LAA | 426 |
| 35 | BAC14510 | sugar ABC transporter | 20 | LAA | 442 |
| 36 | BAC14516 | maltose:maltodextrin transport | 21 | LVA | 425 |
| 37 | BAC14526 | multiple sugar-binding transport | 25 | IAG | 432 |
| 38 | BAC14528 | ribose ABC transporter | 18 | LAA | 303 |
| 39 | BAC15380 | sugar ABC transporter substrate-binding protein | 19 | LVA | 418 |
|  |  | **Unknown transportation** |  |  |  |
| 40 | BAC12387 | ABC transporter permease | 22 | LAA | 313 |
| 41 | BAC12516 | ABC transporter | 20 | LAA | 288 |
| 42 | BAC14272 | ABC transporter substrate-binding protein | 19 | LSA | 328 |
| 43 | BAC14403 | TAXI family TRAP transporter solute-binding subunit | 19 | ITA | 323 |
| 44 | BAC14818 | TRAP transporter substrate-binding protein | 24 | LTS | 357 |
| 45 | BAC14897 | efflux RND transporter periplasmic adaptor subunit | 19 | LAA | 287 |
| 46 | BAC14898 | ABC transporter | 18 | LAA | 331 |
| 47 | BAC15022 | TAXI family TRAP transporter solute-binding subunit | 21 | LAA | 330 |
| 48 | BAC15344 | ABC transporter permease | 21 | LAA | 374 |
| 49 | BAC15430 | ABC transporter | 22 | LVA | 438 |
|  |  | **Cell-Wall** |  |  |  |
| 50 | BAC12786 | N-acetylmuramoyl-L-alanine amidase | 19 | LVA | 234 |
| 51 | BAC13316 | peptidyl-prolyl cis-trans isomerase B (ppiase B) (rotamase B) | 19 | LSA | 202 |
| 52 | BAC14487 | penicillin-binding protein 3 | 17 | LSA | 670 |
|  |  | **Germination** |  |  |  |
| 53 | BAC12153 | spore germination protein | 21 | LTA | 214 |
| 54 | BAC12437 | Ger(x)C family spore germination protein | 18 | LAG | 370 |
| 55 | BAC12652 | spore germination protein | 18 | LAG | 384 |
| 56 | BAC13061 | YhcN/YlaJ family sporulation lipoprotein | 17 | LVA | 188 |
| 57 | BAC14063 | germination (cortex hydrolysis) and sporulation (stage II, multiple polar septa) protein | 20 | LSG | 352 |
| 58 | BAC14722 | Ger(x)C family spore germination protein | 21 | LSG | 368 |
|  |  | **Enzymes and foldases** |  |  |  |
| 59 | BAC12177 | adhesion protein Adp | 17 | LSA | 417 |
| 60 | BAC12500 | SurA N-terminal domain-containing protein | 18 | LVA | 242 |
| 61 | PRSA_OCEIH | foldase protein PrsA | 19 | LSA | 299 |
| 62 | YIDC_OCEIH | Foldase YidC | 20 | LSG | 252 |
| 63 | BAC14295 | superoxide dismutase (Cu-Zn) | 18 | LSA | 195 |
| 64 | BAC12194 | Protease inhibitor I9 family protein | 19 | ISG | 166 |
| 65 | BAC12486 | carboxypeptidase | 19 | ISA | 339 |
| 66 | BAC12717 | CamS family sex pheromone protein | 17 | LSG | 369 |
| 67 | BAC13159 | transcriptional activator of comK gene | 18 | LSA | 320 |
| 68 | BAC13353 | SCO family protein | 16 | LVG | 190 |
| 69 | BAC14619 | class F sortase | 15 | LVA | 217 |
| 70 | BAC14639 | MBL fold metallo-hydrolase | 17 | LIG | 359 |
| 71 | BAC14698 | alpha/beta fold hydrolase | 15 | LVA | 436 |
|  |  | **Unknown function** |  |  |  |
| 72 | BAC12252 | hypothetical protein | 18 | LIG | 158 |
| 73 | BAC12258 | hypothetical conserved protein | 17 | LTG | 123 |
| 74 | BAC12280 | hypothetical protein | 20 | LTG | 136 |
| 75 | BAC12577 | hypothetical conserved protein, outer membrane lipoprotein carrier protein LolA | 22 | LAA | 347 |
| 76 | BAC12727 | hypothetical protein | 19 | LVG | 110 |
| 77 | BAC12897 | hypothetical protein | 19 | LAA | 203 |
| 78 | BAC13338 | hypothetical conserved protein | 18 | LTA | 154 |
| 79 | BAC13342 | hypothetical protein | 20 | LVA | 248 |
| 80 | BAC13367 | YkyA family protein | 20 | ITG | 227 |
| 81 | BAC13373 | hypothetical protein | 19 | LTA | 139 |
| 82 | BAC13683 | hypothetical protein | 19 | LIG | 108 |
| 83 | BAC13995 | hypothetical protein | 19 | LIG | 188 |
| 84 | BAC14041 | hypothetical conserved protein | 20 | LTG | 177 |
| 85 | BAC14047 | extracellular solute-binding protein | 21 | LTA | 504 |
| 86 | BAC14054 | lipoprotein | 18 | TAC | 274 |
| 87 | BAC14322 | hypothetical conserved protein | 16 | LGA | 180 |
| 88 | BAC14404 | hypothetical conserved protein | 18 | LAA | 166 |
| 89 | BAC14612 | hypothetical protein | 17 | LAG | 146 |
| 90 | BAC14699 | hypothetical conserved protein | 18 | LVA | 162 |
| 91 | BAC14728 | hypothetical protein | 18 | LSA | 238 |
| 92 | BAC14732 | hypothetical protein | 19 | LIS | 205 |
| 93 | BAC14884 | hypothetical protein | 18 | LAA | 143 |
| 94 | BAC14901 | hypothetical protein | 20 | ITA | 77 |
| 95 | BAC14947 | hypothetical protein | 23 | VIG | 100 |
| 96 | BAC15069 | hypothetical protein | 18 | ISG | 619 |
| 97 | BAC15166 | hypothetical protein | 21 | LSA | 544 |
| 98 | BAC15202 | hypothetical conserved protein, YehR family protein | 24 | LAA | 153 |
| 99 | BAC13294 | PepSY domain-containing protein | 19 | LIA | 197 |

**S6. Lpp of *Listeria monocytogenes***

| **No** | **Locus tag** | **Function/Annotation** | **SPII (aa)** | **Lipobox** | **Mass (KDa)** |
| --- | --- | --- | --- | --- | --- |
|  |  | **Fe transportation** |  |  |  |
| 1 | NP_463896 | EfeM/EfeO family lipoprotein: ion take-up system | 19 | VVG | 386 |
| 2 | NP_465483 | ferrichrome-binding protein | 20 | LTA | 313 |
| 3 | NP_465708 | ferrichrome ABC transporter | 17 | LVG | 245 |
| 4 | NP_465954 | ferrichrome ABC transporter | 19 | LAS | 323 |
|  |  | **Other cation transportation** |  |  |  |
| 5 | NP_463686 | zinc ABC transporter | 20 | LAG | 313 |
| 6 | NP_464598 | metal ABC transporter | 18 | LTA | 299 |
| 7 | NP_465372 | metal ABC transporter | 18 | LAG | 308 |
|  |  | **Amino acid and peptide transportation** |  |  |  |
| 8 | NP_463668 | peptide ABC transporter | 19 | LTA | 524 |
| 9 | NP_463685 | peptide ABC transporter | 21 | LTA | 551 |
| 10 | NP_463816 | MetQ/NlpA family ABC transporter substrate-binding protein | 22 | LAA | 273 |
| 11 | NP_464541 | glycine/betaine ABC transporter | 20 | LAA | 300 |
| 12 | NP_464951 | glycine/betaine ABC transporter | 22 | LSS | 308 |
| 13 | NP_465720 | peptide ABC transporter substrate-binding protein | 22 | LVA | 558 |
| 14 | NP_465872 | amino acid ABC transporter | 20 | LSA | 269 |
| 15 | NP_466092 | peptide ABC transporter | 22 | LTA | 553 |
|  |  | **Sugar transportation** |  |  |  |
| 16 | NP_463712 | sugar ABC transporter | 20 | LSA | 418 |
| 17 | NP_464295 | sugar ABC transporter | 19 | LAA | 417 |
| 18 | NP_464385 | sugar ABC transporter | 22 | LTA | 439 |
| 19 | NP_465649 | sugar ABC transporter | 22 | LAA | 419 |
| 20 | NP_466361 | sugar ABC transporter | 21 | LAA | 430 |
|  |  | **Unknown transportation** |  |  |  |
| 21 | NP_464069 | ABC transporter | 22 | LTA | 306 |
| 22 | NP_465196 | ABC transporter | 21 | LTG | 317 |
| 23 | NP_465940 | ABC transporter | 19 | LTA | 276 |
|  |  | **Cell wall** |  |  |  |
| 24 | NP_466334 | D-alanyl-D-alanine carboxypeptidase | 19 | LSA | 272 |
|  |  | **Enzymes and foldases** |  |  |  |
| 25 | NP_464904 | Membrane protein insertase YidC | 21 | LTG | 275 |
| 26 | NP_466376 | Membrane protein insertase YidC | 26 | LSG | 287 |
| 27 | NP_464470 | competence protein ComEC, MBL fold metallo-hydrolase | 18 | LSG | 376 |
| 28 | NP_464969 | foldase, peptidylprolyl isomerase PrsA | 21 | LAG | 294 |
| 29 | NP_465282 | CamS family sex pheromone protein | 17 | LSG | 371 |
| 30 | NP_465743 | foldase, peptidylprolyl isomerase PrsA | 20 | LAA | 293 |
| 31 | NP_466101 | alpha/beta hydrolase | 20 | LSA | 291 |
| 32 | NP_466159 | protein FMN transferase | 19 | VSA | 360 |
| 33 | NP_466160 | FMN-binding protein | 22 | LVG | 299 |
| 34 | NP_466165 | metallophosphoesterase | 19 | LVA | 443 |
| 35 | NP_464045 | phosphoglycerate mutase | 20 | IAG | 271 |
| 36 | NP_463885 | fumarate reductase subunit A | 21 | IAG | 506 |
|  |  | **Unknown function** |  |  |  |
| 37 | NP_463580 | hypothetical protein lmo0047, PepSY domain-containing protein | 20 | LLV | 203 |
| 38 | NP_463738 | hypothetical protein lmo0207, YehR family protein | 22 | LVA | 153 |
| 39 | NP_463786 | hypothetical protein lmo0255, YehR family protein | 19 | VTA | 167 |
| 40 | NP_463834 | putaive secreted, lysin rich protein | 19 | LVG | 184 |
| 41 | NP_463854 | hypothetical protein lmo0324 | 21 | LSA | 159 |
| 42 | NP_464038 | hypothetical protein lmo0510 | 17 | LAS | 192 |
| 43 | NP_464144 | hypothetical protein lmo0617 | 22 | LTA | 164 |
| 44 | NP_464318 | hypothetical protein lmo0791 | 20 | LSA | 216 |
| 45 | NP_464348 | hypothetical protein lmo0821 | 18 | LAG | 220 |
| 46 | NP_464790 | hypothetical protein lmo1265 | 19 | LTA | 221 |
| 47 | NP_465174 | hypothetical protein lmo1649 | 19 | LAA | 171 |
| 48 | NP_465531 | hypothetical protein lmo2007 | 20 | LSA | 485 |
| 49 | NP_465603 | hypothetical protein lmo2079 | 20 | ISA | 381 |
| 50 | NP_465604 | hypothetical protein lmo2080 | 20 | LAG | 126 |
| 51 | NP_465855 | hypothetical protein lmo2331 | 21 | LTG | 226 |
| 52 | NP_465939 | hypothetical protein lmo2416 | 20 | LSG | 358 |
| 53 | NP_466117 | hypothetical protein lmo2594 | 18 | VTA | 119 |

**S7. Lpp of *Streptococcus pyogenes***

| **No** | **Locus tag** | **Function/Annotation** | **SPII (aa)** | **Lipobox** | **Mass (KDa)** |
| --- | --- | --- | --- | --- | --- |
|  |  | **Fe transportation** |  |  |  |
| 1 | NP_268705 | ferrichrome ABC transporter | 19 | LIA | 310 |
|  |  | **Other cation transportation** |  |  |  |
| 2 | NP_268747 | metal binding protein of ABC transporter | 20 | LVA | 310 |
| 3 | NP_269968 | metal ABC transporter substrate-binding | 19 | IAG | 306 |
|  |  | **Anion transportation** |  |  |  |
| 4 | NP_269379 | putative phosphate ABC transporter | 22 | LSA | 288 |
|  |  | **Amino acid and peptide transportation** |  |  |  |
| 5 | NP_268656 | amino acid ABC transporter | 22 | LAA | 280 |
| 6 | NP_268657 | MetQ/NlpA family ABC transporter | 21 | LVA | 281 |
| 7 | NP_269403 | putative amino acid ABC transporter | 21 | LVA | 278 |
|  |  | **Sugar transportation** |  |  |  |
| 8 | NP_268607 | putative sugar transporter sugar binding lipoprotein | 21 | LAA | 439 |
| 9 | NP_269421 | putative maltose/maltodextrin-binding protein | 23 | LVG | 415 |
| 10 | NP_269430 | maltose/maltodextrin-binding protein | 24 | LTA | 419 |
|  |  | **Unknown transportation** |  |  |  |
| 11 | NP_268997 | ABC transporter | 21 | LAA | 270 |
| 12 | NP_269366 | BMP family protein | 20 | LAA | 350 |
| 13 | NP_269652 | putative ABC transporter substrate binding lipoprotein | 22 | LAA | 481 |
| 14 | NP_269807 | putative ABC transporter | 19 | LVA | 294 |
| 15 | NP_269961 | ABC transporter substrate-binding protein | 22 | LVA | 542 |
|  |  | **Cell wall** |  |  |  |
| 16 | NP_269064 | putative peptidoglycan hydrolase | 18 | LAA | 235 |
|  |  | **Enzymes and foldases** |  |  |  |
| 17 | NP_268679 | membrane protein insertase YidC | 23 | LTG | 307 |
| 18 | NP_269488 | putative protease maturation protein | 22 | LAA | 351 |
| 19 | NP_269625 | TlpA family protein disulfide reductase | 19 | LTA | 207 |
| 20 | NP_269874 | putative acid phosphatase | 21 | VTG | 284 |
| 21 | NP_268750 | putative cyclophilin-type protein, peptidylprolyl isomerase | 19 | LSA | 268 |
|  |  | **Unknown functions** |  |  |  |
| 22 | NP_269984 | hypothetical protein SPy_2037 | 22 | LSA | 309 |
| 23 | NP_268860 | hypothetical protein SPy_0604 | 19 | LVA | 128 |
| 24 | NP_269417 | hypothetical protein SPy_1290 | 21 | LVA | 206 |
| 25 | NP_269467 | putative internalin A precursor | 24 | LIA | 792 |
| 26 | NP_269499 | hypothetical protein SPy_1405 | 24 | LVG | 75 |

**S8. Lpp of *Streptococcus pneumonia***

| **No** | **Locus tag** | **Function/Annotation** | **SPII (aa)** | **Lipobox** | **Mass (KDa)** |
| --- | --- | --- | --- | --- | --- |
|  |  | **Other cation transportation** |  |  |  |
| 1 | AAK75729 | manganese ABC transporter | 19 | LVA | 309 |
| 2 | ADCA_STRPN | Zinc-binding lipoprotein AdcA | 18 | LVA | 501 |
|  |  | **Anion transportation** |  |  |  |
| 3 | PSTS2_STRPN | Phosphate-binding protein PstS 2 | 21 | LVA | 291 |
|  |  | **Amino acid and peptide transportation** |  |  |  |
| 4 | AAK74331 | MetQ/NlpA family ABC transporter substrate-binding protein | 22 | LAA | 284 |
| 5 | AAK74534 | oligopeptide ABC transporter, oligopeptide-binding protein AliA | 23 | LAA | 661 |
| 6 | AAK74772 | putative amino acid ABC transporter | 16 | LVA | 266 |
| 7 | AAK74888 | branched-chain amino acid ABC transporter | 20 | LAA | 386 |
| 8 | AAK75492 | amino acid ABC transporter | 21 | LVA | 271 |
| 9 | AAK75591 | amino acid ABC transporter | 19 | LVA | 278 |
| 10 | ALIB_STRPN | Oligopeptide-binding protein Ali | 24 | LSA | 652 |
| 11 | AMIA_STRPN | Oligopeptide-binding protein AmiA | 22 | LAA | 659 |
|  |  | **Sugar transportation** |  |  |  |
| 12 | AAK75762 | sugar ABC transporter | 21 | LAA | 442 |
| 13 | MALX_STRPN | Maltose/maltodextrin-binding protein | 24 | LVA | 423 |
|  |  | **Unknown transportation** |  |  |  |
| 14 | AAK74976 | lipoprotein, BMP family protein | 21 | VAA | 350 |
| 15 | AAK74279 | ABC transporter | 23 | LAA | 491 |
| 16 | AAK74330 | ABC transporter | 23 | LAA | 276 |
| 17 | AAK75769 | ABC transporter | 22 | LVA | 445 |
| 18 | AAK75869 | ABC transporter | 22 | LAA | 538 |
| 19 | AAK75899 | ABC transporter | 22 | LAA | 355 |
| 20 | AAK76248 | putative ABC transporter | 22 | LVA | 335 |
|  |  | **Enzymes and foldases** |  |  |  |
| 21 | AAK74780 | metallopeptidase | 18 | LAA | 238 |
| 22 | AAK74804 | thioredoxin family protein | 19 | LTA | 188 |
| 23 | AAK74909 | peptidyl-prolyl cis-trans isomerase | 17 | LAG | 267 |
| 24 | PRSA_STRPN | Foldase protein PrsA | 20 | LAA | 313 |
| 25 | YIDC1_STRPN | Membrane protein insertase YidC | 22 | LTG | 308 |
|  |  | **Unknown functions** |  |  |  |
| 26 | AAK74372 | hypothetical protein SP_0191 | 17 | LVG | 189 |
| 27 | AAK75025 | conserved hypothetical protein | 22 | LAA | 290 |

**S9. Lpp of *Streptococcus agalactiae***

| **No** | **Locus tag** | **Function/Annotation** | **SPII (aa)** | **Lipobox** | **Mass (KDa)** |
| --- | --- | --- | --- | --- | --- |
|  |  | **Other cation transportation** |  |  |  |
| 1 | CAD47236 | nickel ABC transporter | 19 | LTA | 538 |
|  |  | **Anion transportation** |  |  |  |
| 2 | PSTS1_STRA3 | Phosphate-binding protein PstS 1 | 22 | LSG | 288 |
| 3 | PSTS2_STRA3 | Phosphate-binding protein PstS 2 | 23 | LAG | 293 |
|  |  | **Amino acid and peptide transportation** |  |  |  |
| 4 | CAD45789 | peptide ABC transporter | 26 | LAA | 551 |
| 5 | CAD46596 | amino acid ABC transporter | 21 | LSA | 276 |
| 6 | CAD46966 | laminin-binding surface protein | 19 | IAG | 306 |
| 7 | CAD47348 | amino acid ABC transporter | 22 | LAA | 277 |
|  |  | **Unknown transportation** |  |  |  |
| 8 | CAD45829 | ABC transporter | 22 | LVA | 542 |
| 9 | CAD45878 | osmoprotectant ABC transporter | 22 | ISG | 308 |
| 10 | CAD46084 | BMP family ABC transporter | 20 | LTA | 347 |
| 11 | CAD46601 | BMP family ABC transporter | 20 | LAA | 349 |
| 12 | CAD46625 | ABC transporter | 28 | LVA | 531 |
| 13 | CAD47122 | ABC transporter | 18 | LVS | 310 |
| 14 | CAD47160 | transporter substrate-binding | 20 | LVA | 268 |
| 15 | CAD47291 | ABC transporter | 20 | LAA | 388 |
| 16 | CAD47318 | transporter substrate-binding | 19 | LAA | 285 |
|  |  | **Enzymes and foldases** |  |  |  |
| 17 | YIDC1_STRA3 | Membrane protein insertase YidC 1 | 20 | LVA | 271 |
| 18 | YIDC2_STRA3 | Membrane protein insertase YidC 2 | 23 | LTG | 310 |
| 19 | PRSA_STRA3 | Foldase protein PrsA | 22 | LAA | 309 |
| 20 | CAD47245 | peptidylprolyl isomerase B | 19 | LSG | 267 |
|  |  | **Unknown functions** |  |  |  |
| 21 | CAD45731 | Unknown | 20 | LAG | 62 |
| 22 | CAD46041 | hypothetical protein | 21 | LAA | 160 |
| 23 | CAD46117 | hypothetical protein | 20 | LVA | 118 |
| 24 | CAD46422 | DUF4430 domain-containing protein | 19 | LVA | 129 |
| 25 | CAD46577 | pneumococcal-type histidine triad protein | 22 | LTA | 877 |
| 26 | CAD46875 | DUF3862 domain-containing protein | 22 | LTA | 207 |
| 27 | CAD47148 | prealbumin-like fold domain-containing protein | 18 | LVG | 577 |

**S10. Lpp of *Streptococcus mutans***

| **No** | **Locus tag** | **Function/Annotation** | **SPII (aa)** | **Lipobox** | **Mass (KDa)** |
| --- | --- | --- | --- | --- | --- |
|  |  | **Anion transportation** |  |  |  |
| 1 | AE014951_1 | putative ABC transporter, phosphate-binding protein | 20 | LAA | 287 |
|  |  | **Sugar transportation** |  |  |  |
| 2 | MSME_STRMU | Multiple sugar-binding protein | 22 | LAA | 420 |
| 3 | AE014988_2 | putative maltose/maltodextrin ABC transporter | 23 | LVA | 415 |
|  |  | **Amino acid and peptide transportation** |  |  |  |
| 4 | AE014922_6 | putative amino acid transporter | 22 | LAA | 271 |
| 5 | AE014922_8 | putative amino acid transporter | 21 | LVA | 271 |
| 6 | AE014954_7 | putative ABC transporter, glutamine binding protein | 21 | LTA | 277 |
| 7 | AE014996_11 | putative ABC transporter, branched chain amino acid-binding protein | 20 | LAA | 390 |
| 8 | AE015018_6 | putative amino acid binding protein | 22 | LTA | 267 |
|  |  | **Unknown transportation** |  |  |  |
| 9 | AE014909_1 | putative ABC transporter | 25 | LVA | 342 |
| 10 | AE014958_6 | putative ABC transporter | 20 | LVA | 283 |
| 11 | AE015033_6 | putative ABC transporter; osmoprotectant-binding protein, glycine betaine/carnitine/choline ABC transporter | 21 | LTS | 311 |
|  |  | **Enzymes and foldases** |  |  |  |
| 12 | YIDC1_STRMU | Membrane protein insertase YidC 1 | 20 | LSA | 271 |
| 13 | PRSA_STRMU | Foldase protein PrsA | 21 | LAA | 333 |
| 14 | AE014994_1 | putative peptidyl-prolyl cis-trans isomerase | 17 | LVG | 258 |
| 15 | YIDC2_STRMU | Membrane protein insertase YidC 2 | 23 | LSG | 310 |
| 16 | AE015018_5 | O-sialoglycoprotein endopeptidase | 22 | LTA | 280 |
|  |  | **Unknown functions** |  |  |  |
| 17 | AE014874_8 | hypothetical protein SMU_252 | 20 | VTA | 257 |
| 18 | AE014912_8 | hypothetical protein SMU_690 | 22 | LIG | 195 |
| 19 | AE014914_2 | conserved hypothetical protein | 19 | LIA | 129 |
| 20 | AE015028_5 | hypothetical protein SMU_2061 | 20 | LVA | 217 |

**S11. Lpp of *Enterococcus faecalis***

| **No** | **Locus tag** | **Function/Annotation** | **SPII (aa)** | **Lipobox** | **Mass (KDa)** |
| --- | --- | --- | --- | --- | --- |
|  |  | **Fe transportation** |  |  |  |
| 1 | NP_813989 | iron-hydroxamate ABC transporter substrate-binding protein | 23 | LAA | 312 |
| 2 | NP_815350 | iron ABC transporter substrate-binding protein | 21 | LAG | 319 |
| 3 | NP_816693 | siderophore ABC transporter substrate-binding protein | 21 | LSA | 317 |
|  |  | **Other cation transportation** |  |  |  |
| 4 | NP_813865 | zinc ABC transporter substrate-binding protein | 23 | LAS | 317 |
| 5 | NP_815118 | molybdate ABC transporter substrate-binding protein | 22 | IAA | 266 |
| 6 | NP_815739 | metal ABC transporter substrate-binding protein | 19 | LAA | 308 |
|  |  | Anion transportation |  |  |  |
| 7 | NP_815411 | phosphate ABC transporter substrate-binding protein | 19 | LTG | 296 |
| 8 | NP_815462 | phosphate ABC transporter substrate-binding protein PstS | 19 | LTG | 284 |
|  |  | **Amino acid and peptide transportation** |  |  |  |
| 9 | NP_813871 | peptide ABC transporter substrate-binding protein | 22 | LAA | 559 |
| 10 | NP_814550 | peptide ABC transporter substrate-binding protein | 19 | LAA | 543 |
| 11 | NP_814605 | glycine betaine/carnitine/choline ABC transporter | 21 | LAG | 307 |
| 12 | NP_814645 | peptide ABC transporter substrate-binding protein | 22 | LAA | 556 |
| 13 | NP_814793 | peptide ABC transporter substrate-binding protein | 18 | LTA | 553 |
| 14 | NP_814849 | amino acid ABC transporter substrate-binding domain-containing | 22 | IVG | 275 |
| 15 | NP_815234 | peptide ABC transporter substrate-binding protein | 19 | LVG | 550 |
| 16 | NP_815491 | peptide ABC transporter substrate-binding protein | 20 | LAG | 546 |
| 17 | NP_815743 | MetQ/NlpA family ABC transporter substrate-binding protein | 20 | IAG | 277 |
| 18 | NP_816357 | peptide ABC transporter substrate-binding protein | 21 | LSG | 542 |
| 19 | NP_816653 | peptide ABC transporter substrate-binding protein | 20 | LAA | 551 |
| 20 | NP_816692 | peptide ABC transporter substrate-binding protein | 19 | LAA | 547 |
| 21 | NP_816716 | oligopeptide ABC transporter substrate-binding protein | 22 | LAA | 593 |
|  |  | **Sugar transportation** |  |  |  |
| 22 | NP_815066 | sugar ABC transporter sugar-binding protein | 24 | LTA | 416 |
| 23 | NP_815895 | sugar ABC transporter substrate-binding protein | 23 | LTA | 425 |
|  |  | **Unknown transportation** |  |  |  |
| 24 | NP_813979 | BMP family ABC transporter substrate-binding protein | 22 | LAA | 357 |
| 25 | NP_813980 | BMP family ABC transporter substrate-binding protein | 22 | LAA | 361 |
| 26 | NP_814214 | TRAP transporter substrate-binding protein | 22 | LSG | 335 |
| 27 | NP_814961 | ABC transporter substrate-binding protein | 19 | LAA | 532 |
| 28 | NP_815882 | ABC transporter substrate-binding protein | 22 | LSA | 488 |
|  |  | **Enzymes and foldases** |  |  |  |
| 29 | NP_816926 | membrane protein insertase YidC | 22 | LSA | 275 |
| 30 | NP_814292 | thermonuclease family protein | 22 | LSG | 194 |
| 31 | NP_814435 | foldase, peptidylprolyl isomerase | 20 | LAA | 342 |
| 32 | NP_815061 | peptidase M4 | 21 | LAS | 166 |
| 33 | NP_815252 | peptidyl-prolyl cis-trans isomerase B | 21 | LAG | 249 |
| 34 | NP_816359 | alpha/beta hydrolase | 22 | LSA | 292 |
| 35 | NP_816590 | alkaline phosphatase | 19 | LAG | 471 |
| 36 | NP_816853 | FMN-binding protein, pheromone cAD1 lipoprotein | 22 | LAA | 309 |
|  |  | **Unknown functions** |  |  |  |
| 37 | NP_813898 | Lpp | 21 | LAG | 261 |
| 38 | NP_813925 | hypothetical protein | 16 | LTA | 125 |
| 39 | NP_813965 | Lpp | 19 | VSG | 172 |
| 40 | NP_814096 | Lpp | 20 | LSA | 176 |
| 41 | NP_814165 | Lpp UF4822 domain-containing protein | 22 | IAG | 325 |
| 42 | NP_814282 | Lpp DUF4923 family protein | 20 | LSG | 114 |
| 43 | NP_814391 | hypothetical protein | 19 | LAA | 305 |
| 44 | NP_814392 | DUF1307 domain-containing protein | 19 | LAS | 149 |
| 45 | NP_814768 | Lpp DUF4767 domain-containing protein | 18 | LSS | 217 |
| 46 | NP_814944 | Lpp | 16 | LTG | 134 |
| 47 | NP_815083 | hypothetical protein | 20 | VSA | 255 |
| 48 | NP_815311 | Lpp | 21 | LTG | 336 |
| 49 | NP_815537 | hypothetical protein | 18 | LVG | 138 |
| 50 | NP_815807 | Lpp | 20 | LTA | 286 |
| 51 | NP_815898 | Lpp | 16 | LTG | 128 |
| 52 | NP_816155 | Lpp | 19 | LTA | 119 |
| 53 | NP_816421 | DUF4950 domain-containing protein, LysM domain-containing protein | 19 | LTS | 354 |
| 54 | NP_816618 | Lpp | 15 | LTG | 230 |
| 55 | NP_816666 | LPP | 19 | VAG | 208 |
| 56 | NP_816800 | membrane protein, YaeC family lipoprotein | 19 | LAA | 272 |
| 57 | NP_816832 | Lpp DUF5105 domain-containing protein | 18 | LAS | 239 |
| 58 | NP_814771 | Lpp | 18 | LVG | 361 |
| 59 | NP_814816 | hypothetical protein | 24 | LSG | 80 |

**S12. Lpp of *Clostridium perfingens***

| **No** | **Locus tag** | **Function/Annotation** | **SPII (aa)** | **Lipobox** | **Mass (KDa)** |
| --- | --- | --- | --- | --- | --- |
|  |  | **Fe transportation** |  |  |  |
| 1 | BAB80144 | ion-uptake ABC transporter | 23 | LAG | 350 |
|  |  | **Anion transportation** |  |  |  |
| 2 | BAB80343 | phosphate ABC transporter | 22 | LTG | 272 |
|  |  | **Amino acid and peptide transportation** |  |  |  |
| 3 | BAB80306 | amino acid ABC transporter | 24 | LAG | 278 |
| 4 | BAB80975 | spermidine/putrescine-binding protein 1 | 21 | LVG | 348 |
| 5 | BAB81030 | amino acid ABC transporter | 20 | ISG | 262 |
| 6 | BAB81799 | amino acid ABC transporter | 21 | LVG | 502 |
|  |  | **Sugar transportation** |  |  |  |
| 7 | BAB80077 | sugar ABC transporter substrate-binding protein | 19 | LAG | 422 |
| 8 | BAB81047 | galactoside ABC transporter | 21 | LVG | 354 |
| 9 | BAB82049 | maltose ABC transporter | 24 | LVG | 408 |
|  |  | **Unknown transportation** |  |  |  |
| 10 | BAB80924 | ABC transporter | 22 | LAG | 333 |
| 11 | BAB80963 | ABC transporter substrate-binding protein | 24 | LAG | 483 |
| 12 | BAB81015 | BMP family ABC transporter substrate-binding protein | 22 | IAG | 356 |
| 13 | BAB81193 | ABC transporter substrate-binding protein | 23 | LVG | 334 |
| 14 | BAB81784 | ABC transporter substrate-binding protein | 24 | LTG | 482 |
|  |  | **Germination** |  |  |  |
| 15 | BAB80355 | spore germination protein B3 | 22 | LIG | 374 |
|  |  | **Enzymes and foldases** |  |  |  |
| 16 | BAB79957 | phosphoserine phosphatase | 23 | LVS | 440 |
| 17 | BAB80432 | cyclically-permuted mutarotase family protein | 19 | LAG | 384 |
| 18 | BAB80756 | 5'-methylthioadenosine/S-adenosylhomocysteine nuclosidase | 23 | LIA | 266 |
| 19 | BAB81273 | peptidyl-prolyl cis-trans isomerase B | 23 | LIG | 210 |
| 20 | BAB81812 | thiamine biosynthesis lipoprotein | 21 | LIS | 343 |
| 21 | BAB80753 | thioredoxin family protein | 19 | LVG | 149 |
|  |  | **Unknown functions** |  |  |  |
| 22 | BAB80133 | hypothetical protein | 19 | LIA | 192 |
| 23 | BAB80139 | WG repeat-containing protein | 22 | LTG | 431 |
| 24 | BAB80240 | hypothetical protein | 22 | LVG | 176 |
| 25 | BAB80729 | DUF4883 family protein | 21 | LIS | 156 |
| 26 | BAB80736 | ABC transporter substrate-binding protein | 24 | LTG | 482 |
| 27 | BAB80845 | hypothetical protein | 19 | LVG | 264 |
| 28 | BAB81221 | DUF4358 domain-containing protein | 20 | LTS | 165 |
| 29 | BAB81235 | DUF2334 domain-containing protein | 19 | LIG | 445 |
| 30 | BAB81260 | hypothetical protein | 20 | LVG | 281 |
| 31 | BAB81277 | hypothetical protein | 22 | LVG | 233 |
| 32 | BAB81286 | membrane lipoprotein TmpC precursor | 21 | LAG | 370 |
| 33 | BAB81551 | hypothetical protein | 21 | LAG | 343 |
| 34 | BAB82219 | hypothetical protein | 21 | IAG | 279 |
| 35 | BAB82261 | hypothetical protein | 20 | LVG | 147 |
| 36 | BAB82310 | GerMN domain-containing protein | 17 | LVG | 184 |

**S13. Lpp of *Mycobacterium tuberculorsis***

| **No** | **Locus tag** | **Function/Annotation** | **SPII (aa)** | **Lipobox** | **Mass (KDa)** |
| --- | --- | --- | --- | --- | --- |
|  |  | **Other cation transportation** |  |  |  |
| 1 | MODA_MYCTO | Molybdate-binding protein ModA | 21 | LVA | 261 |
|  |  | **Anion transportation** |  |  |  |
| 2 | PSTS2_MYCTO | Phosphate-binding protein PstS | 22 | LTA | 370 |
|  |  | **Amino acid and peptide transportation** |  |  |  |
| 3 | AAK44648 | amino acid ABC transporter | 25 | LAS | 328 |
| 4 | AAK48131 | peptide ABC transporter | 24 | VAG | 541 |
|  |  | **Sugar transportation** |  |  |  |
| 5 | LPRG_MYCTO | Lipoprotein LprG, lipoarabinomannan carrier protein | 26 | VAG | 236 |
|  |  | **Lipid transportation** |  |  |  |
| 6 | LPQW_MYCTO | Monoacyl phosphatidylinositol tetramannoside-binding protein LpqW | 27 | LAG | 635 |
| 7 | LPPX_MYCTO | Phthiocerol dimycocerosate transporter LppX | 26 | LSG | 233 |
|  |  | **Enzymes and foldases** |  |  |  |
| 8 | LPRI_MYCTO | Lipoprotein LprI, MliC family protein, membrane bound lysozyme inhibitor | 15 | LSA | 197 |
| 9 | AAK45984 | thiol:disulfide interchange protein | 19 | VTA | 182 |
| 10 | AAK46759 | gamma-glutamyltransferase | 20 | LSG | 643 |
| 11 | AAK44469 | beta-N-acetylhexosaminidase | 19 | VAA | 388 |
| 12 | SODC_MYCTO | Superoxide dismutase (SOD) | 32 | LSA | 240 |
| 13 | AAK47740 | esterase | 18 | LAG | 304 |
|  |  | **Unknown functions** |  |  |  |
| 14 | AAK44856 | hypothetical protein MT0632, DUF1259 domain-containing protein | 22 | LTA | 316 |
| 15 | LPRP_MYCTO | Lipoprotein LprP | 21 | LAG | 224 |
| 16 | LPQV_MYCTO | Lipoprotein LpqV | 25 | VAG | 139 |
| 17 | AAK45540 | Lipoprotein LpqZ | 22 | VAG | 286 |
| 18 | LPRH_MYCTO | Lipoprotein LprH, sensor domain-containing protein | 27 | LAA | 228 |
| 19 | LPRA_MYCTO | Lipoprotein LprA | 24 | IGG | 244 |
| 20 | AAK46244 | Lipoprotein LppF | 20 | VIG | 423 |
| 21 | AAK46296 | virulence factor mce family protein | 16 | VAS | 377 |
| 22 | AAK46480 | Lipoprotein LppL | 25 | VAG | 358 |
| 23 | LPPN_MYCTO | Lipoprotein LppN | 20 | LAA | 175 |
| 24 | AAK46771 | Lipoprotein LppR, sensor domain-containing protein | 19 | AAG | 251 |
| 25 | MP83_MYCTO | Cell surface glycolipoprotein MPT83 | 24 | LAG | 220 |
| 26 | LPPW_MYCTO | Lipoprotein LppW | 22 | VAG | 314 |
| 27 | LPQB_MYCTO | Lipoprotein LpqB | 19 | LAG | 587 |
| 28 | AAK47958 | virulence factor mce family protein | 20 | LAG | 384 |
| 29 | AAK48086 | Lipoprotein LpqG, SIMPL domain-containing protein | 22 | LSG | 240 |
| 30 | LPQH_MYCTO | Lipoprotein LpqH | 21 | LSG | 195 |
